# FOXA2/miR-148a-3p/SMURF2 signaling feed-forward loop alleviates spinal cord ischemia-reperfusion injury-induced neuropathic pain by modulating microglia polarization in rats

**DOI:** 10.3389/fimmu.2025.1563377

**Published:** 2025-05-13

**Authors:** Xiaotong Hao, Linyan Cao, Jinshi Li, Qian Lei, Xuan Liu, Yuanyuan Li, Yiting Fan, Jingjing Xu, Bo Fang

**Affiliations:** Department of Anesthesiology, The First Hospital of China Medical University, Shenyang, Liaoning, China

**Keywords:** smurf2, SIRT1, miR-148a-3p, foxa2, spinal cord ischemia-reperfusion injury, neuropathic pain, microglia polarization

## Abstract

**Background:**

Microglia polarization is crucial in mediating neuropathic pain. However, the role of microglia polarization in regulating spinal cord ischemia-reperfusion injury (SCIRI)-induced neuropathic pain is largely unknown. This study aimed to elucidate the relationship between SCIRI-induced neuropathic pain and microglia polarization, as well as the underlying mechanisms, with the objective of identifying potential therapeutic targets.

**Methods:**

A rat model of SCIRI was established by aortic arch clamping, then pain thresholds were measured. *In vitro*, oxygen-glucose deprivation/reperfusion (OGD/R) of HAPI microglia was performed. The expressions of sirtuin1 (SIRT1), SMAD specific E3 ubiquitin protein ligase 2 (SMURF2), and markers of microglial polarization (CD206, iNOS) were quantitated by Western blot and immunofluorescence, and the levels of cytokines (TNF-α, IL-4) were assessed by Enzyme-linked immunosorbent assay (ELISA). Real-time quantitative reverse transcription PCR (RT-qPCR) experiments were conducted to quantify the levels of miR-148a-3p and FOXA2. Dual-luciferase reporter assay was employed to identify the targeted regulation of SMURF2 by miR-148a-3p and the transcriptional regulation of miR-148a-3p by FOXA2. The regulatory role of FOXA2 in the transcription of miR-148a-3p was validated using chromatin immunoprecipitation (ChIP). In addition, co-immunoprecipitation (Co-IP) assays were performed to confirm the binding relationship between SMURF2 and FOXA2. And the ubiquitination levels of FOXA2 and SIRT1 were measured. Subsequently, rats were administered miR-148a-3p to assess pain thresholds. Western blot and immunofluorescence quantitative analysis was conducted to assess the expression of markers associated with microglia polarization.

**Results:**

SCIRI significantly reduced mechanical and thermal pain thresholds in rats and promoted microglial polarization, with a concomitant decrease in SIRT1 expression and an increase in SMURF2 expression in microglial cells. Further analysis revealed that upregulation of miR-148a-3p promoted microglia polarization toward M2 by targeting SMURF2, which in turn inhibited ubiquitination of SIRT1. FOXA2 was an upstream transcription factor of miR-148a-3p and SMURF2 bound to FOXA2, resulting in its ubiquitination. Finally, *in vivo* experiments demonstrated that miR-148a-3p effectively promoted microglia transformation from M1 to M2 and reduced neuropathic pain following SCIRI.

**Conclusions:**

The FOXA2/miR-148a-3p/SMURF2 signaling feed-forward loop regulates SIRT1 levels and thereby exerts control over microglia polarization and the regulation of SCIRI-induced neuropathic pain.

## 1 Introduction

Spinal cord ischemia-reperfusion injury (SCIRI) represents a significant adverse consequence of thoracoabdominal aortic surgery and spinal cord decompression surgery (1). Previous studies have concentrated on SCIRI-induced motor pathologies, such as paraplegia and paralysis (2). Nevertheless, it is imperative to recognize that neuropathic pain is also a prominent symptom of SCIRI, which can significantly impair the quality of life of patients (3, 4). Neuropathic pain, is a pathological condition triggered by injuries or diseases impacting the somatosensory system, presents symptoms including allodynia and hyperalgesia (5, 6). Recent research has emphasized the critical role of microglia in the progression of neuropathic pain. For instance, microglia, which lead to the degradation of the network around neurons, and microglia chemokines, all influence the occurrence of neuropathic pain (7, 8). As resident immune sentinels in the central nervous system (9), microglia exhibit remarkable phenotypic plasticity. Timothy R. et al. identified a microglial subpopulation that exhibited distinctive inflammatory signaling characteristics, as determined by single-cell sequencing techniques. This subpopulation may play a specific role in initiating inflammatory responses (10). Under pathological conditions, microglia undergo polarization into two classical phenotypes: the pro-inflammatory M1-type (characterized by inducible nitric oxide synthase (iNOS) pression) that exacerbates neural damage through tumor necrosis factor-α (TNF-α) release (11–13), and the anti-inflammatory M2-type (marked by CD206) that promotes neural repair via protective mediators like interleukin-4 (IL-4) (14–16). It is important to acknowledge that, subsequent to SCIRI, in addition to the classical M1/M2 phenotype (17), there are also intermediate M1/2 microglia with dual phenotypic characteristics (18). A number of signaling factors and compounds have been demonstrated to modulate neuropathic pain by modulating M1/M2 polarization. These include TRAF6 (19), Huangqin declination (20), and DUSP1 (21), botulinum toxin (22), among others. These studies suggest that this dynamic phenotypic shift in M1/M2 may constitute an important regulatory node for the development of neuropathic pain (23).

Mammalian cell sirtuin1 (SIRT1) is a kind of deacetylase dependent on nicotinamide adenosine dinucleotide (NAD+). SIRT1 has been known to be involved in neuroinflammation, which can inhibit microglia activation and other inflammatory reactions (24–26), and relieve neuropathic pain through a variety of signal pathways, such as the NF-KB, p53 and PI3K/AKT (4, 27, 28). In addition, overexpression of SIRT1 promotes microglia to M2 type polarization (29). SMAD ubiquitin regulatory factor 2 (SMURF2) has been shown to function as an E3 ubiquitin ligase, associated with post-ischemic neural damage (30, 31) and inflammation (32, 33), and regulates protein expression in macrophages (34). Recent evidence reported that SMURF2 has a specific binding affinity for SIRT1 and induces its ubiquitin-mediated degradation (35). However, the potential involvement of SIRT1 is poorly elucidated in neuropathic pain.

Specific miRNAs have been the subject of considerable interest due to their capacity to influence pain perception. For example, the miRNA-22/MTF1 signaling axis has been identified as a key player in the initiation and maintenance of inflammatory pain in the spinal dorsal horn (36). Conversely, miR-32-5p downregulation in trigeminal ganglion neurons has been shown to regulate trigeminal neuropathic pain (37). These findings highlight the complex and multifaceted roles of miRNAs in pain modulation. FOXA is a member of the forkhead box protein family, which includes FOXA1, FOXA2, and FOXA3. It has a wide range of functions, including roles in development, glycolipid metabolism, the aging process, and immune regulation. Despite the existence of numerous reports investigating the interaction between FOXA and miRNAs (38, 39), the functional role of these interactions in neuropathic pain, particularly in SCIRI, has yet to be elucidated.

The aim of this study was to explore the molecular mechanism of neuropathic pain after SCIRI, so as to explore promising therapeutic targets for neuropathic pain.

## 2 Materials and methods

### 2.1 Experimental animals and ethics statement

In this study, Sprague-Dawley (SD) rats weighing between 200–250 g were utilized as the primary experimental animals. These rats were procured from the Experimental Animal Center of the China Medical University (Shenyang, China). The animals were housed in standard cages under controlled environmental conditions: a temperature of 22 ± 2°C, humidity levels maintained at 50 ± 10%, and a 12 h/12 h light/dark cycle. Before initiating the experiments, the rats were given a week-long acclimatization period where they were allowed ad libitum access to both food and water. All surgical interventions were conducted under anesthesia to minimize pain and distress to the animals. The experimental protocols, especially those involving animal handling and procedures, were approved by the Animal Care and Use Committee of China Medical University.

### 2.2 Rat model of SCIRI

The aortic cross-clamping method was utilized to induce SCIRI (40). Rats were anesthetized through intraperitoneal injection of 4% pentobarbital sodium at a dose of 50 mg/kg. The rectal temperature was diligently maintained at 37 ± 0.5°C throughout the surgical procedure using a heating lamp. Once anesthetized, rats were positioned in lateral recumbency, and the aortic arch was exposed via a cervicothoracic approach. Clamping was executed between the left common carotid artery and the left subclavian artery, sustained for a period of 8 minutes.

### 2.3 Intrathecal injection

A total of four days prior to the administration of SCIRI, rats were anesthetized with 3% isoflurane. An intrathecal catheter was then placed in the L5-L6 intervertebral space. Injections were administered at 12 h intervals between each, and the injections spanned three consecutive days. Each injection encompassed 10 μl of either miR-148a-3p mimic or NC mimic, at a concentration of 50 μmol/L with invitrorna™ (InvivoGene Biotechnology, Nanjing, China).

### 2.4 Nociceptive behavioral testing

Prior to the commencement of the experiment, the rats were acclimatized to the environment for a period of one hour. Mechanical sensitivity was assessed using the paw withdrawal threshold (PWT), defined as the force (g) causing the rat to retract its paws. PWT was measured using von Frey filaments (Anesthesio, Danmic Global, USA), which were gently lifted upward perpendicular to the sole of the mouse until the filaments were bent. Positive responses, indicated by avoidance movements such as retraction, shaking, or licking of the affected limb, were recorded. The measurements were repeated four times at this stimulus intensity, and the average PWT was calculated.

Thermal sensitivity was assessed 15 minutes after the PWT measurement using the paw withdrawal latency (PWL) method. PWL denotes the duration taken for a rat to conspicuously retract its paw when placed on a hot plate (YAN-6B, YUYAN Shanghai, China) set at 50°C. To prevent potential tissue damage, the maximum exposure time on the hot plate was capped at 25 seconds. This measurement was taken three times with a 10 minutes interval between each to compute the average PWL. All behavioral tests were administered in a blinded manner.

### 2.5 Experimental protocols

#### 2.5.1 Protocol I

**Figure 1:**
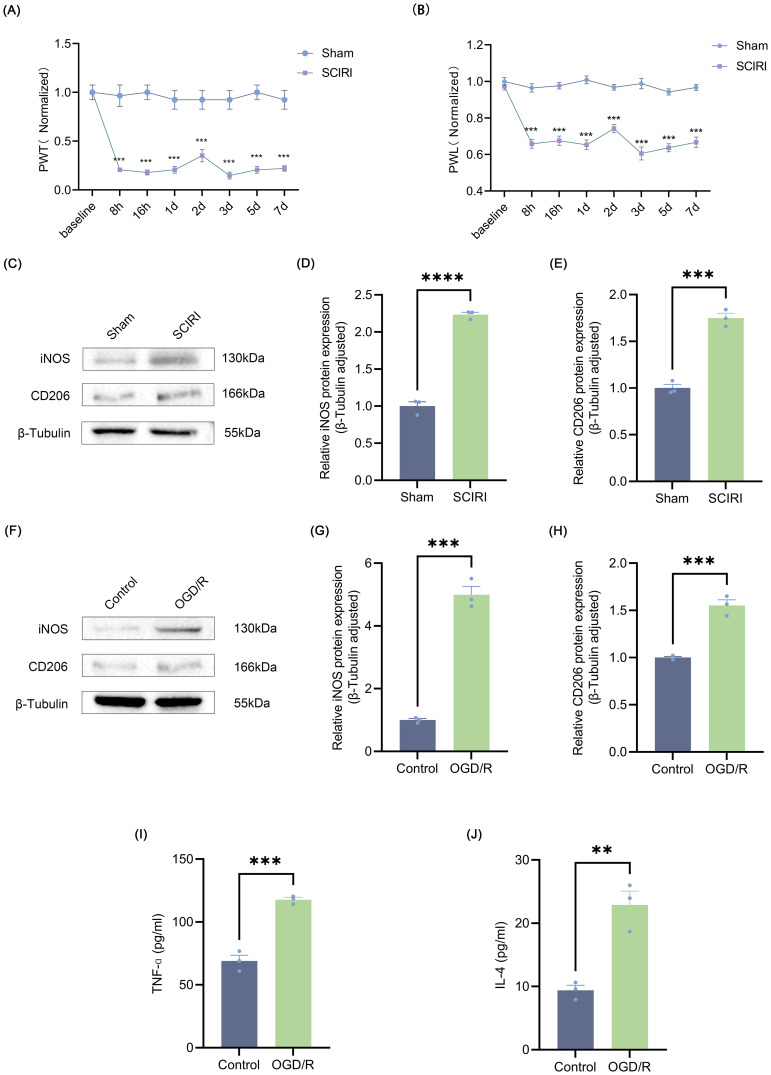
SCIRI induces neuropathic pain and microglia polarization. **(A, B)** Changes in PWT and PWL after SCIRI in rats. Data are expressed as normalized values against their respective Sham. **(C)** Representative Western blot bands representing temporal expression changes of iNOS and CD206 in the spinal cord of rats following SCIRI. **(D, E)** Quantitative protein analysis of iNOS and CD206 in the spinal cord of rats following SCIRI. **(F)** Representative Western blot of iNOS and CD206. **(G, H)** Quantitative protein analysis of iNOS and CD206 in the HAPI following OGD/R. **(I, J)** HAPI cells supernatant TNF-α and IL-4 are detected by ELISA. Repeated-measures analysis of variance was utilized in **(A, B)** n=6 in each group. Unpaired t-test was utilized. Significance levels: **P<0.01, ***P<0.001, ****P<0.0001.

**Figure 2:**
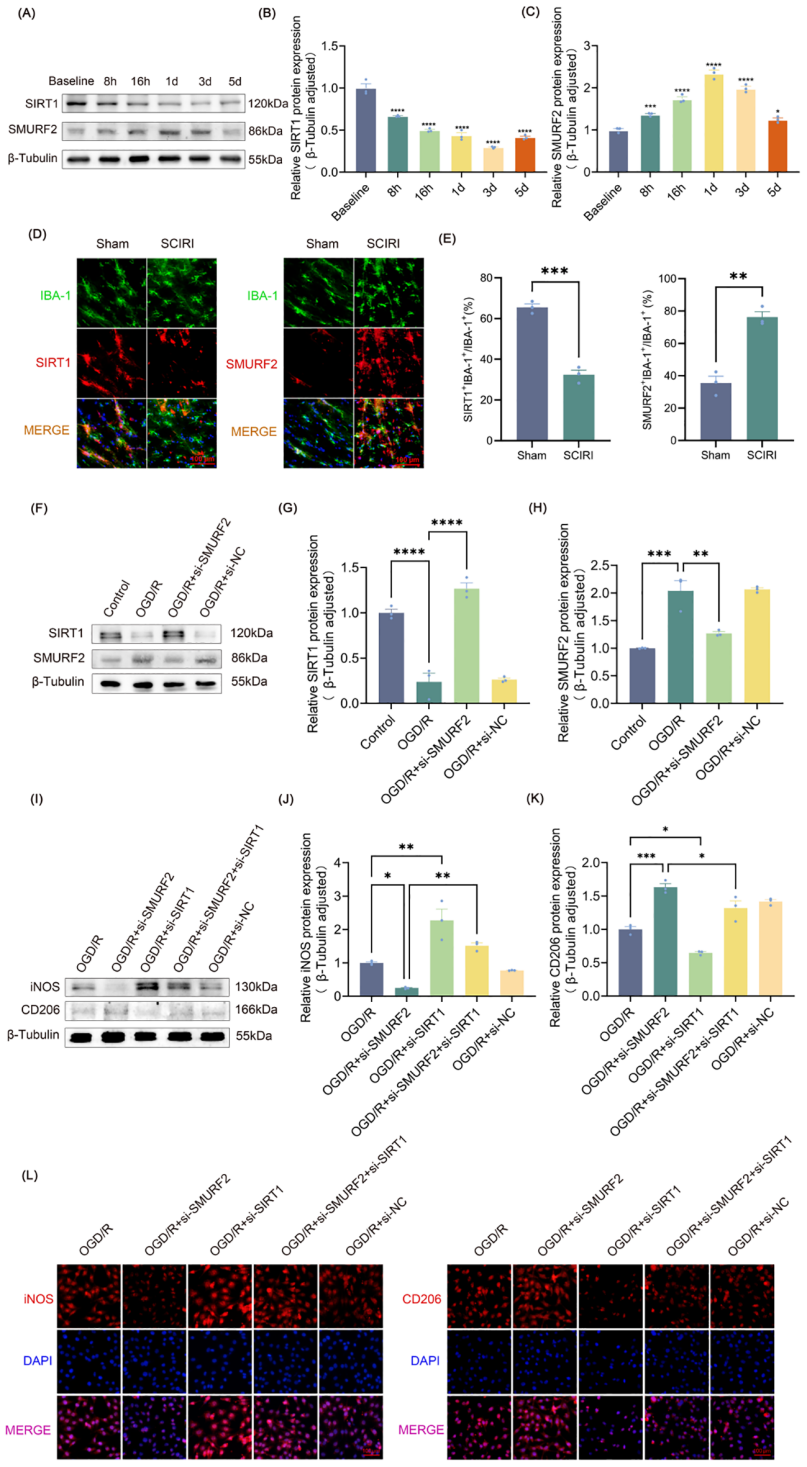
SMURF2 regulates microglia polarization by downregulating SIRT1 after SCIRI. **(A)** Representative Western blot bands representing temporal expression changes of SIRT1 and SMURF2 in the spinal cord of rats following SCIRI. **(B, C)** Quantitative protein analysis of SIRT1 and SMURF2 in the spinal cord of rats following SCIRI. **(D)** Immunofluorescence revealed the co-expression of SIRT1 or SMURF2 and IBA-1 in the rat spinal cord three days after SCIRI. Scale bar: 100 μm. **(E)** Quantitative fluorescence area analysis. **(F)** Representative Western blot of SIRT1 and SMURF2. **(G, H)** Quantitative protein analysis of SIRT1 and SMURF2. **(I)** Representative Western blot of iNOS and CD206. **(J, K)** Quantitative protein analysis of iNOS and CD206. **(L)** Immunofluorescence revealed iNOS-positive and CD206-positive microglial cells. Scale bar: 100 μm. Unpaired t-test and one-way ANOVA were utilized. Significance levels: *P<0.05, **P<0.01, ***P<0.001, ****P<0.0001.

Rats were randomly divided into a Sham group (n = 6) and a SCIRI group (n = 6). The SCIRI group underwent the SCIRI procedure, while the sham group underwent the same procedure except that the aorta was clamped. PWT and PWL were measured in the hindpaw 6 hours before SCIRI and 8 hours, 16 hours, and on days 1, 2, 3, 5, and 7 after SCIRI. Additionally, rats were euthanized at 6 hours before SCIRI and at 8 hours, 16 hours, as well as on days 1, 3, and 5 after SCIRI to obtain spinal cord tissue for Western blot analysis. Spinal cord tissue on day 3 after SCIRI was used for immunofluorescence analysis (**Figures 1**, **2**).

#### 2.5.2 Protocol II

**Figure 3:**
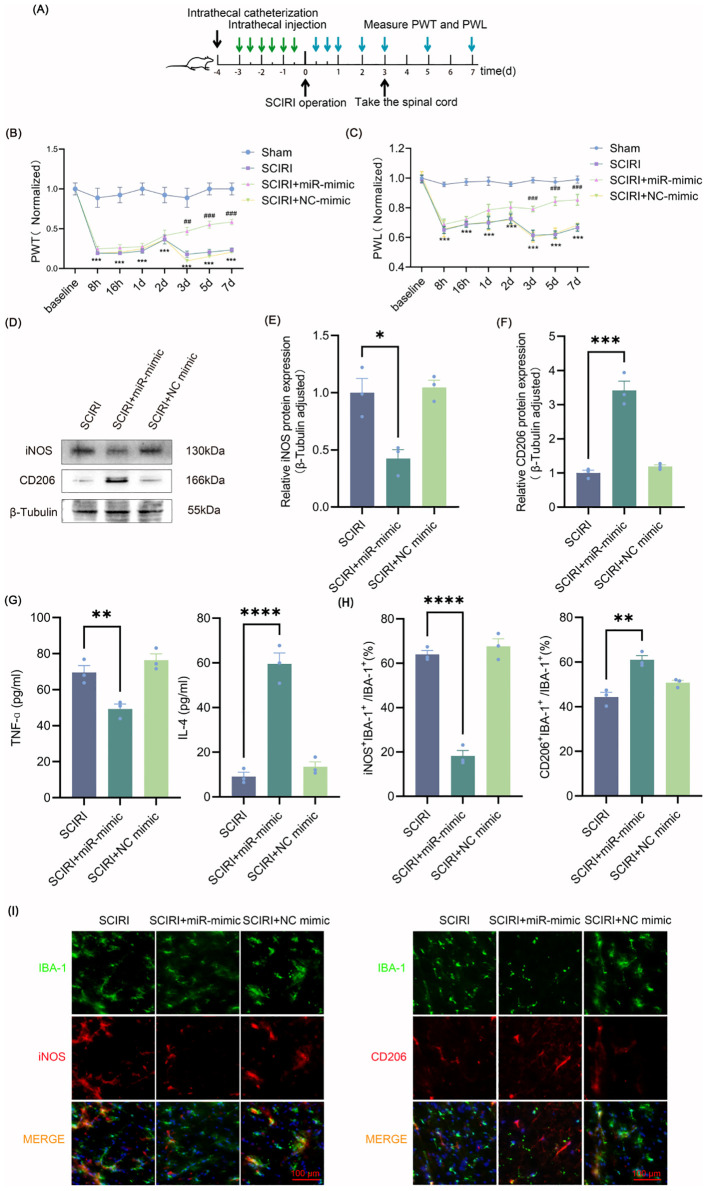
Intrathecal injection of miR-148a-3p polarizes spinal microglia to M2 and relieves neuropathic pain after SCIRI. **(A)** Process schematic. **(B, C)** Changes of PWT and PWL over time in rats. Data are expressed as normalized values against their respective Sham. **(D)** Representative Western blot of iNOS and CD206 in the spinal cords of rats. **(E, F)** Quantitative protein analysis of iNOS and CD206. **(G)** Evaluation of microglia polarization markers by ELISA. **(H)** Quantitative fluorescence area analysis. **(I)** Immunofluorescence co-localization revealed iNOS-positive and CD206-positive microglial cells. Scale bar: 100 μm. Repeated-measures analysis of variance was utilized in **(B, C)** n=6 in each group. One-way ANOVA was used in E-H. Significance levels: *P<0.05, **P<0.01, ***P<0.001, ****P<0.0001. ***p<0.001 Sham versus SCIRI, and ##p<0.01, ###p<0.001 SCIRI versus SCIRI+miR-mimic in **(B, C)**.

Rats were divided into 4 groups (n = 6 rats/group): sham; SCIRI; SCIRI treated with miR-mimic (SCIRI + miR-mimic); SCIRI treated with NC mimic (SCIRI + NC mimic). PWT and PWL were assessed in the hindpaw 6 hours before SCIRI and 8 hours, 16 hours, and days 1, 2, 3, 5, and 7 after SCIRI. Rats in the SCIRI, SCIRI + miR-mimic, and SCIRI + NC mimic groups were euthanized on day 3 post-SCIRI for Western blot analysis, ELISA, and immunofluorescence (**Figure 3**).

### 2.6 Cell culture and transfection

HAPI, HEK293T and VSC4.1 cells were cultured in Dulbecco’s Modified Eagle Medium (DMEM) obtained from Procell Company, Wuhan, China. The culture medium was supplemented with 10% Fetal Bovine Serum (FBS) and 100 U/ml of penicillin/streptomycin. Cells were maintained according to the supplier’s recommendations. For transfection experiments, cells were transfected with small interfering RNA using invitroRNA™ (InvivoGene Biotechnology, Nanjing, China) following the manufacturer’s guidelines.

### 2.7 Oxygen-glucose deprivation/reperfusion

HAPI cells were cultured in a serum-free and glucose-free DMEM medium and then placed into a sterile three-gas incubator (94%N_2_, 5%CO_2_, 1%O_2)_, incubated at 37°C for 4 h to induce OGD injury. The cells were then returned to a 37°C, 5% CO_2_ incubator and incubated in a normal medium for a 24 h reperfusion period. The control HAPI was grown in a normoxic incubator in a medium containing glucose for the same time period.

### 2.8 Western blot

The extracted spinal cord tissue from the expanded segment (L4-6) of rats and cell samples were meticulously placed in 1.5mL EP tubes. The protein plasma was collected by means of centrifugation after protein extraction using Radioimmunoassay (RIPA) lysis buffer. The proteins were then separated using Sodium Dodecyl Sulfate-Polyacrylamide Gel Electrophoresis (SDS-PAGE) with a 10% gel. The transfer was executed onto a PVDF membrane (EMD Millipore, Bedford, USA). The membranes were then incubated with the following primary antibodies: anti-SMURF2 at a dilution of 1:800 (Proteintech, Wuhan, China), anti-SIRT1 at 1:1000 (Abcam, Shanghai, China), anti-iNOS and anti-CD206 both at 1:500 (Abmart, Shanghai, China), anti-FOXA2 at 1:1000 (Abcam, Cambridge, UK), and anti-β-Tubulin at 1:10,000 (Abmart, Shanghai, China). Incubation with primary antibodies was performed on a shaker at 4°C overnight. This was followed by a 1 h incubation with the secondary antibodies, either horseradish peroxidase-conjugated anti-mouse or anti-rabbit antibodies (Abmart, Shanghai, China). For semi-quantitative analysis of the protein bands, the Quantity One software (Bio-Rad Laboratories, Milan, Italy) was utilized.

### 2.9 Enzyme-linked immunosorbent assay

Spinal cord tissue was washed with pre-chilled saline, cut into small pieces, and homogenized by adding pre-chilled RIPA protein lysate containing PMSF. Following centrifugation, the supernatant was separated for cytokine testing. HAPI cells were disrupted, and centrifuged, and the supernatant collected for cytokine testing. TNF-α and IL-4 levels were determined using specific ELISA kits (Nanjing Jiancheng Bioengineering, China) according to the manufacturer’s instructions. Absorbance was measured at a wavelength of 450 nanometers using a microplate reader. The average values were calculated using a three-hole testing method.

### 2.10 Immunofluorescence

The spinal cords from the L4-L6 segments were sectioned and fixed in 4% paraformaldehyde for a period of 2–4 h. Thereafter, the tissues were cryopreserved overnight at 4°C in 30% sucrose phosphate buffer. Subsequently, the tissues were cut into sections 10 μm thick using a Leica CM 3050s cryostat (Leica Biosystems, USA). To block non-specific binding, the sections were incubated in 10% bovine serum albumin (BSA) for 1 h at room temperature. For immunofluorescence staining, the sections were then incubated overnight at 4°C with primary antibodies, including anti-iNOS, anti-CD206, anti-SMURF2, anti-SIRT1, and anti-IBA-1 (1:150, Novus, Shanghai, China). Following this, the sections were incubated with secondary antibodies: Dylight 594-affinipure donkey anti-rabbit IgG (1:500, Boster, Wuhan, China) and Alexa 488-conjugated donkey anti-Goat IgG (1:500, Abcam, Shanghai, China) for 1 h at room temperature, followed by staining with DAPI for 5 minutes. For cell samples, the fixed cells were permeabilized following a 4% paraformaldehyde incubation and blockade at room temperature, cells were incubated overnight with primary antibodies anti-iNOS and anti-CD206 (1:150, Shanghai, China) and stained with DAPI after incubation with secondary antibodies for 1 h at room temperature. Finally, images were captured using a universal fluorescence microscope (Nicon, Tokyo, Japan) equipped with an attached digital camera.

### 2.11 Cell counting kit-8 assay

Cells were seeded in 96-well plates at a density of 5 × 10³ cells/well in 100 μL of complete culture medium and incubated overnight at 37°C under 5% CO_2_ to allow adherence. After 24 hours of treatment with miR-mimic, NC mimic, or control, 10 μL of CCK-8 reagent was added to each well, and the plates were further incubated for 2 hours at 37°C. Absorbance was measured at 450 nm using the enzyme-linked immunosorbent assay reader. Wells containing culture medium and CCK-8 reagent without cells served as blanks. All experiments were performed in quintuplicate.

### 2.12 Real-time quantitative reverse transcription PCR

Total RNA was extracted using an RNAiso Plus reagent (Takara, Beijing, China) as per the manufacturer’s instructions. For cDNA synthesis, 1 µg of RNA was reverse-transcribed using the PrimeScript™ RT reagent Kit with gDNA Eraser (Takara, Beijing, China). Quantitative real-time PCR (RT-qPCR) analysis was carried out using TB GREEN Premix Ex Taq™ II (Takara, Beijing, China) on a standard qPCR system. GAPDH and U6 were used as internal reference genes. The data were calculated by the 2−ΔΔCt method, and the experiment was repeated three times independently. The primer sequences used for amplification are provided in **Supplementary Table S1**.

### 2.13 Dual-luciferase reporter assay

In order to verify SMURF2 targeting by miR-148a-3p, the SMURF2 wild-type 3′ untranslated region (3′UTR) containing the miR-148a-3p binding site and the SMURF2 mutant 3′UTR were serially inserted into the pmirGLO vector. Subsequently, HEK293T cells were seeded in 24-well plates at a density of 2×104 per well and cultured to 60% confluency. Subsequently, the cells were cotransfected with the recombinant plasmid (wild-type/mutant) and miR-148a-3p mimic or negative control (NC) mimic 24 hours later, along with Renilla luciferase vector as an internal control. Luciferase activity was measured 48 hours after transfection using the Dual-Luciferase Reporter Assay System (Promega, Beijing, China) according to the manufacturer’s instructions.

In order to investigate the effect of FOXA2 on miR-148a-3p transcriptional activity, wild-type miR-148a-3p promoter and FOXA2 binding site mutants were inserted into the pGL3-Basic vector. The resulting constructs were then co-transfected into HEK293T cells with pcDNA3.1-FOXA2 overexpressing constructs or empty vectors. Luciferase activity was measured 48 hours after transfection. Firefly luciferase activity was normalized to Renilla luciferase activity to account for transfection efficiency.

### 2.14 Co-immunoprecipitation

The FOXA2 antibody or SMURF2 antibody was coupled with protein A/G agarose beads (Takara Biotechnology, Dalian, China) at 4°C for 1 hour. Following this, the cells were washed with pre-chilled phosphate-buffered saline (PBS). Thereafter, the cells were lysed on ice using Pierce IP lysis buffer (Thermo Fisher Scientific) for 30 minutes. The resulting supernatant was then incubated overnight at 4°C with rotation with antibody-conjugated agarose beads. Thereafter, the precipitated complex was washed with wash buffer and eluted with elution buffer. The eluted samples were subsequently analyzed using Western blot. IgG isotype served as the negative control.

### 2.15 Chromatin immunoprecipitation assay

Subsequent to crosslinking the cells with 1% formaldehyde at room temperature for 10 minutes, the reaction was terminated with 125 mM glycine. Thereafter, the cells were lysed to collect the chromatin. The DNA was fragmented using sonication and then co-cubated overnight at 4°C with FOXA2 antibody or isotype-matched control IgG. The incubation period was overnight at 4°C. Antibody-protein-DNA complexes were isolated by immunoprecipitation using Protein A Sepharose beads. Following a thorough washing procedure, the bound DNA fragments were eluted and subsequently analyzed by RT-qPCR. The primer sequences are listed in the **Supplementary Table S1**.

### 2.16 Statistical analysis

Prism GraphPad 9.5.0 and IBM SPSS 27 were utilized for the consolidation and statistical analysis of experimental data, presenting results as means ± SEM. To examine the distribution of the data, the Shapiro-Wilk normality test was used. Differences were evaluated using the unpaired t-test between the two groups if data were normally distributed and homogeneous variance. For comparisons involving multiple groups, one-way ANOVA was utilized. Repeated-measures analysis of variance was used to compare the differences between the two groups at different time points. P < 0.05 indicated statistical significance.

## 3 Results

### 3.1 SCIRI induces neuropathic pain and microglia polarization

To investigate post-SCIRI neuropathic pain, PWT and PWL tests were conducted at designated time points following SCIRI or sham operation. It was observed that following SCIRI, rats exhibited a decline in the mechanical pain threshold and a reduction in PWL in the hot plate test. Both the mechanical pain threshold and thermal pain threshold reached their lowest values on the third day (**Figures 1A, B**). These findings indicated that SCIRI triggers neuropathic pain. Prior research has demonstrated that neuropathic pain is inextricably linked to microglial polarization (41). Therefore, we studied the changes in microglia polarization after SCIRI. Spinal cord tissue was collected three days post-surgery, and Western blot was performed to assess the expression levels of the microglia polarization markers iNOS and CD206. The results suggested that SCIRI significantly increased the expression of iNOS and CD206 (**Figures 1C–E**). OGD/R was performed on microglia *in vitro* to simulate SCIRI conditions *in vivo*. Similarly, we observed significant elevations in the expressions of iNOS and CD206 (**Figures 1F–H**). ELISA analysis corroborated the elevated levels of TNF-α, a pro-inflammatory factor associated with M1 polarization, and IL-4, an anti-inflammatory factor associated with M2 polarization (**Figures 1I, J**). Therefore, it is inferred that SCIRI may affect microglia polarization and thus promote the development of neuropathic pain.

### 3.2 SMURF2 regulates microglia polarization by downregulating SIRT1 after SCIRI

It is known that SIRT1 can alleviate neuropathic pain in many ways, and the regulation of microglia polarization is one of the most critical mechanisms (42). It has been demonstrated that SMURF2, which functions as an E3 ubiquitin ligase, could bind to SIRT1 and facilitate its ubiquitin-dependent degradation (35). However, the effect of SMURF2 on microglial polarization is not yet available. The expressions of SIRT1 and SMURF2 in the spinal cord at various time points following SCIRI were measured. SIRT1 expression was progressively reduced post-SCIRI, reaching minimal levels by day three (**Figures 2A, B**). SMURF2 increased as early as 8 h after injury, peaked between 1–3 days, and subsequently decreased on the fifth day (**Figures 2A, C**). The expression trends of SIRT1 and SMURF2 coincided with neuropathic pain in rats after SCIRI, suggesting a potential relationship between these two molecules and post-SCIRI neuropathic pain in rats.

Frozen sections of the L4-L6 spinal cord were obtained from both the sham-operated rats and the rats 3 days after SCIRI. Immunofluorescence staining was performed on the sections using IBA-1 (green) to label activated microglia. And then, the sections were co-labeled with either SIRT1 (red) or SMURF2 (red) (**Figure 2D**). The results demonstrated that the number of IBA-1-positive cells increased in the presence of SCIRI. The ratio of the co-localization area of SIRT1 and IBA-1 to the area of IBA-1 was found to decrease. Conversely, following SCIRI, the ratio of SMURF2 and IBA-1 co-localization area to IBA-1 area in the spinal cord was observed to increase. These outcomes indicated that SCIRI activated spinal microglia and altered the expression of SIRT1 and SMURF2 in microglia. (**Figures 2D, E**). These findings presented that SCIRI activated spinal microglia and changed the expression of SIRT1 and SMURF2 in microglia.

**Figures 2F–H** illustrated that OGD/R increased the expression of SMURF2 and decreased the expression of SIRT1. However, the knockdown of SMURF2 (**Supplementary Figure S1A**) reversed the decrease of SIRT1. To evaluate the impact of SMURF2 on microglia polarization, the expressions of iNOS and CD206 were evaluated by Western blot and immunofluorescence. As shown in **Figures 2I and J**, the reduction in iNOS expression resulting from SMURF2 knockdown can be partially reversed by SIRT1 knockdown (**Supplementary Figure S1B**). Conversely, the knockdown of SMURF2 led to an increase in CD206 expression, while the knockdown of SIRT1 partially reversed the effect of SMURF2 on CD206 levels. The simultaneous knockdown of SMURF2 and SIRT1 resulted in a partial recovery of the elevated CD206 (**Figures 2I, K**). The immunofluorescence experiment provided a more visual representation (**Figure 2L**), which was consistent with the results of the Western blot. Taken together, the above results revealed that SMURF2 regulated microglial polarization via SIRT1 after SCIRI.

### 3.3 miR-148a-3p reduces ubiquitination of SIRT1 by targeting SMURF2

**Figure 4:**
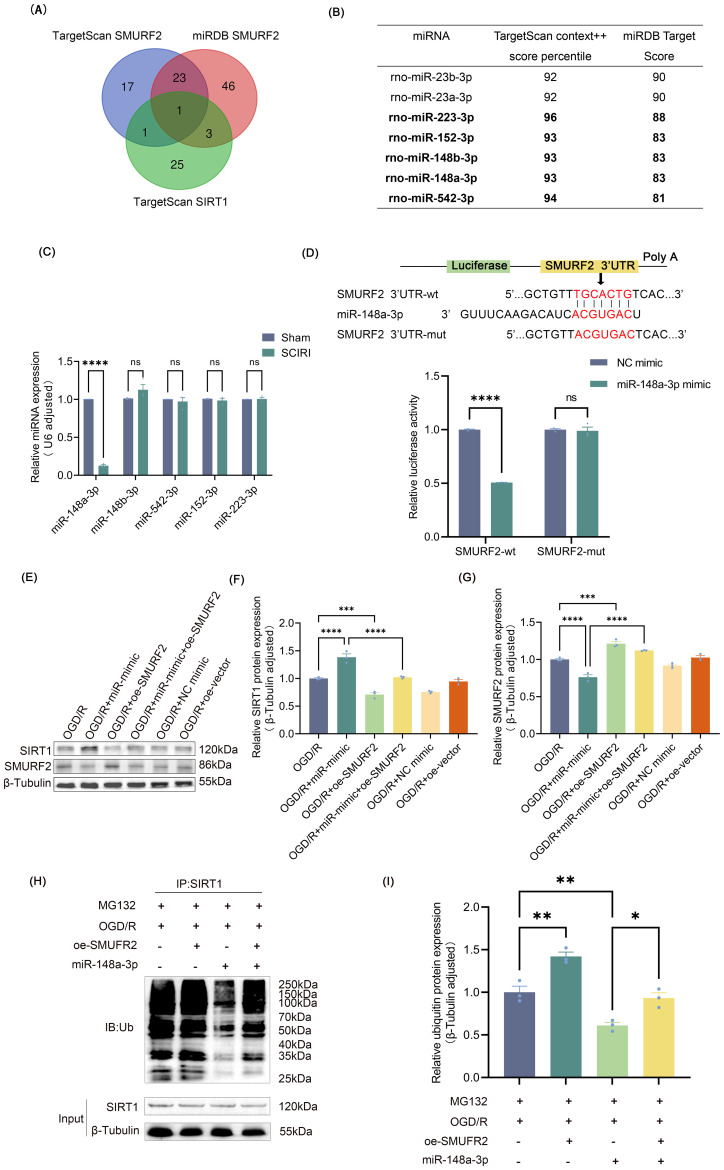
miR-148a-3p reduces ubiquitination of SIRT1 by targeting SMURF2 **(A)** miRNAs predicted by TargetScan 8.0 and miRDB databases. **(B)** The selection criteria included miRNAs with TargetScan scores above 90 and miRDB scores above 80. **(C)** RT-qPCR analysis. **(D)** Dual-luciferase reporter gene assay. **(E)** Representative Western blot of SIRT1 and SMURF2. **(F, G)** Quantitative protein analysis of SIRT1 and SMURF2. **(H)** Immunoprecipitation followed by Western blot was employed to detect the ubiquitination levels of SIRT1. **(I)** Quantitative protein analysis of ubiquitin. Unpaired t-test and one-way ANOVA were utilized. Significance levels: *P<0.05, **P<0.01, ***P<0.001, ****P<0.0001.

Building on our findings, the expression of SMURF2 was elevated in microglia following the induction of SCIRI or OGD/R, and its involvement in regulating microglia polarization was established. miRNAs can modulate neuropathic pain resulting from spinal cord injury by regulating target genes as previously reported (43). To determine potential miRNAs that could target SMURF2, TargetScan 8.0 and miRDB databases were employed to predict upstream miRNAs. After taking the intersection of the predicted results and excluding those that could potentially bind to SIRT1, 23 miRNAs were identified (**Figure 4A**). Seven of the identified miRNAs exhibited TargetScan context++ score percentiles exceeding 90, accompanied by miRDB scores exceeding 80 (**Figure 4B**). In light of prior research, miR-23b-3p and miR-23a-3p inhibited SIRT1 (44, 45), which was inconsistent with the original hypothesis of this study. Subsequently, RT-qPCR was used to determine the expression of five other miRNAs in the injured spinal cord of rats. Only miR-148a-3p was found to decrease after SCIRI (**Figure 4C**). Dual-luciferase assays confirmed that miR-148a-3p mimic obviously reduced the luciferase activity in the group of SMURF2-wt but not in the group of SMURF2-mut (**Figure 4D**). These findings provided evidence that miR-148a-3p could specifically target and bind to the predicted sites in SMURF2.

To investigate the effects and underlying mechanism of miR-148a-3p on SIRT1, plasmids were constructed for overexpressing SMURF2 (**Supplementary Figure S2A**), and miR-mimic was used for overexpressing miR-148a-3p (**Supplementary Figure S2B**). To exclude potential confounding effects of miR-148a-3p mimics on cellular viability, we conducted CCK-8 assays in both HAPI and VSC4.1 cells treated with miR-mimic. The results demonstrated no significant alterations in cell viability compared to control groups (**Supplementary Figures S2C, D**), validating the safety of miR-mimic under these experimental conditions. As is shown in **Figures 4E–G**, overexpression of miR-148a-3p exhibited a decrease in SMURF2 and an increase in SIRT1. However, overexpression of SMURF2 reverted the up-regulation of SIRT1. It suggested that miR-148a-3p could elevate SIRT1 expression by inhibiting SMURF2 expression. Consistent with previous studies, immunoprecipitation and Western blot revealed that overexpression of SMURF2 led to an increased ubiquitination of SIRT1. Meanwhile, miR-148a-3p reduced SIRT1 ubiquitination, and SMURF2 effectively reverted the decreased ubiquitination of SIRT1 induced by miR-148a-3p (**Figures 4H, I**). Collectively, these findings displayed that miR-148a-3p decreased the ubiquitination of SIRT1 by suppressing SMURF2, thereby preventing the degradation of SIRT1.

### 3.4 FOXA2/miR-148a-3p/SMURF2 signaling feed-forward loop regulates microglia polarization

**Figure 5:**
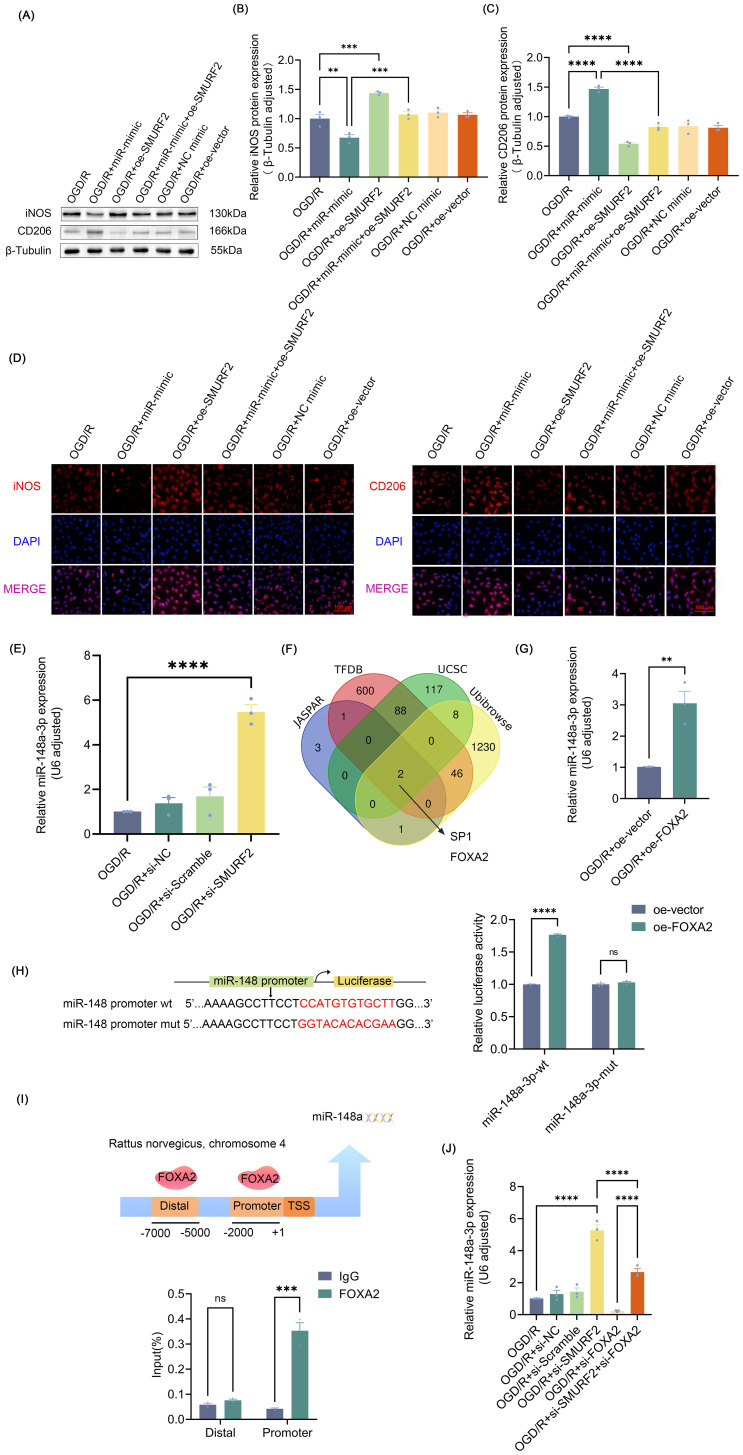
FOXA2/miR-148a-3p/SMURF2 signaling feed-forward loop regulates microglia polarization **(A)** Representative Western blot of iNOS and CD206. **(B, C)** Quantitative protein analysis of iNOS and CD206. **(D)** Immunofluorescence revealed iNOS-positive and CD206-positive microglial cells. Scale bar: 100 μm. **(E)** RT-qPCR analysis. **(F)** JASPAR, UCSC, TFDB, and Ubibrowser 2.0 databases were used to predict the potential transcription factors of miR-148a-3p. **(G)** RT-qPCR analysis. **(H)** Dual-luciferase reporter gene assay. **(I)** The binding of FOXA2 to the miR-148a promoters was tested by ChIP assays. **(J)** RT-qPCR analysis. Unpaired t-test and one-way ANOVA were utilized. Significance levels: *P<0.05, **P<0.01, ***P<0.001, ****P<0.0001.

As shown in **Figures 5A–C**, miR-148a-3p promoted the polarization of M2 microglia, manifesting as an increase in CD206 and a decrease in iNOS, and overexpression of SMURF2 led to the restoration of CD206 and iNOS levels. Immunofluorescence results were consistent with the Western blot findings (**Figure 5D**), suggesting that miR-148a-3p polarizes microglia towards the M2 phenotype by suppressing SMURF2. Interestingly, SMURF2 knockdown significantly increased miR-148a-3p expression levels (**Figure 5E**). Therefore, it is speculated that SMURF2 was not only the downstream target gene of miR-148a-3p but also the regulatory factor of miR-148a-3p. As a broad E3 ubiquitin ligase, SMURF2 regulates the ubiquitination and degradation of numerous proteins including transcription factors. Given this, we were prompted to investigate whether SMURF2 might be involved in the regulation of miR-148a-3p transcription factor. Next, we predicted transcription factors of miR-148a-3p from the JASPAR, UCSC, and TFDB databases, combining them with the ubiquitination substrate of SMURF2 from Ubibrowser 2.0 database. Two potential molecules, SP1 and FOXA2, were selected (**Figure 5F**).

Previous studies have shown that SP1 expression elevated following the onset of SCIRI (46, 47), and that up-regulation of SP1 was found to be significantly correlated with the enhancement of prior pain perception (47), which were at odds with the inference of the present study. Therefore, FOXA2 was identified as a suitable subject for further investigation. The results demonstrated a notable elevation in miR-148a-3p expression levels following FOXA2 overexpression (**Figure 5G**, **Supplementary Figure S3A**). Subsequently, dual-luciferase reporter gene detection and ChIP assays revealed that FOXA2 was bound to the miR-148a-3p promoter region and regulated its transcriptional activity (**Figures 5H, I**). Meanwhile, the knockdown of FOXA2 (**Supplementary Figure S3B**) reversed the effects of SMURF2 on miR-148a-3p (**Figure 5J**), suggesting that SMURF2 regulated miR-148a-3p through FOXA2. Combined with the previously demonstrated regulation of microglia polarization by inhibition of SMURF2 by miR-148a-3p, it can be concluded that FOXA2/miR-148a-3p/SMURF2 signaling feed-forward loop regulates microglial polarization.

### 3.5 SMURF2 promotes FOXA2 ubiquitination and degradation

**Figure 6:**
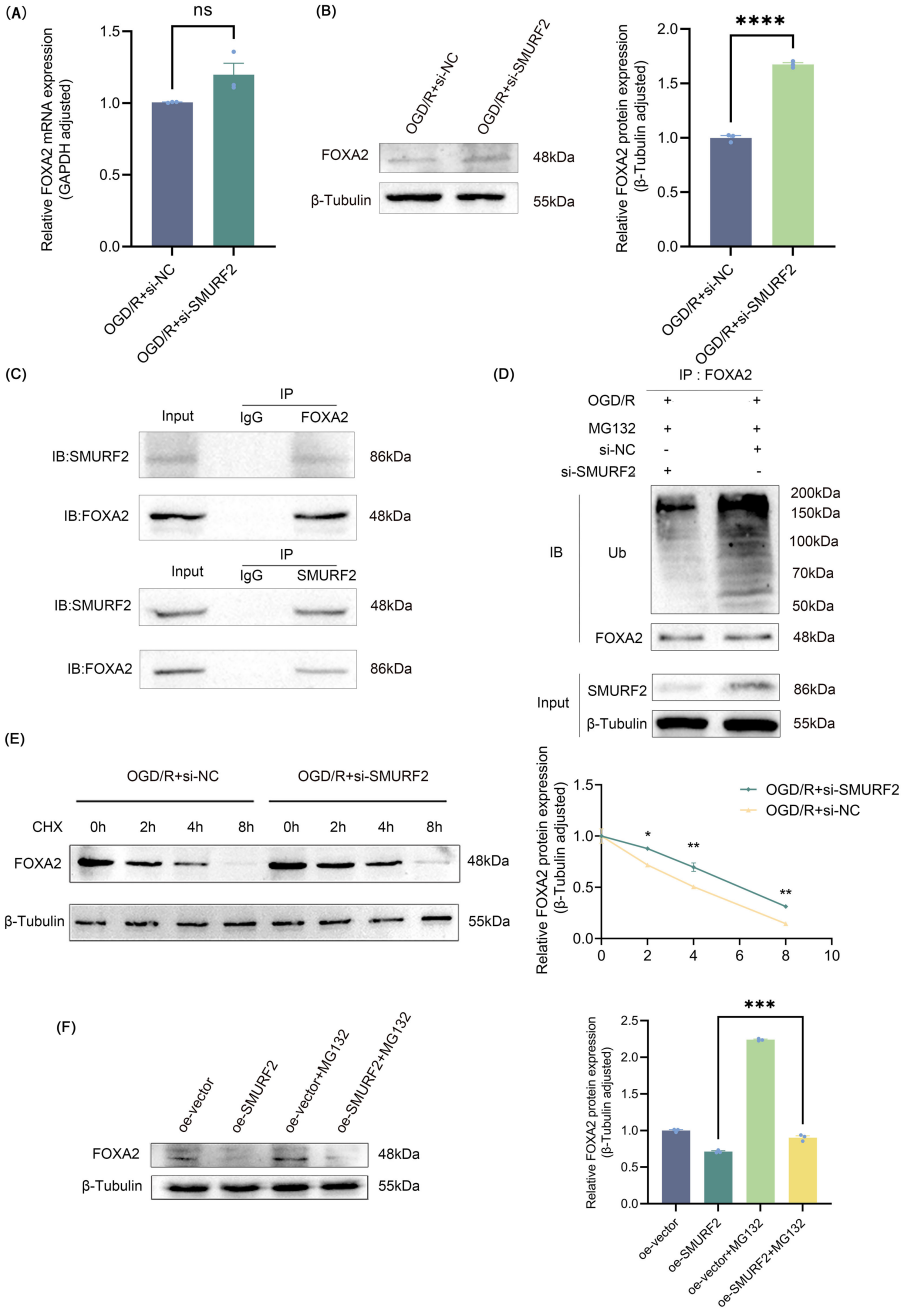
SMURF2 promotes FOXA2 ubiquitination and degradation. **(A)** RT-qPCR analysis. **(B)** Representative Western blot of FOXA2 and quantitative protein analysis of FOXA2. **(C)** Co-IP assay suggested protein-protein interactions between SMURF2 and FOXA2. **(D)** Detection of ubiquitination level of FOXA2 by Co-IP. **(E)** Western blot analysis of FOXA2 protein levels treated with CHX. **(F)** Western blot analysis of FOXA2 protein levels treated with MG132. Unpaired t-test and one-way ANOVA were utilized. Significance levels: *P<0.05, **P<0.01, ***P<0.001, ****P<0.0001, ns indicates no significant difference (P ≥ 0.05).

To investigate how SMURF2 affects FOXA2, the expression changes of FOXA2 were explored at both mRNA and protein levels, respectively, and the results showed that SMURF2 had no effect on FOXA2 mRNA expression, but significantly inhibited FOXA2 protein expression (**Figures 6A, B**). The Co-IP assay further confirmed the interaction between SMURF2 and FOXA2 (**Figure 6C**). Next, we performed ubiquitination validation and found that SMURF2 promoted the ubiquitination of FOXA2, leading to its degradation (**Figure 6D**). By treating cells with the protein translation inhibitor cycloheximide (CHX), it was shown that the degradation rate of FOXA2 was slowed down after SMURF2 knockdown (**Figure 6E**). In addition, the ubiquitination degradation of FOXA2 was also restored by the proteasome inhibitor MG132, further confirming that SMURF2 promoted the ubiquitination degradation of FOXA2 (**Figure 6F**). These results suggested that SMURF2 could reduce FOXA2 protein levels by promoting its ubiquitination and degradation.

### 3.6 Intrathecal injection of miR-148a-3p polarizes spinal microglia to M2 and relieves neuropathic pain after SCIRI

Intrathecal injection of miR-mimic did not improve the mechanical and thermal pain thresholds in the first two days after SCIRI. However, miR-mimic showed beneficial effects on the third day after SCIRI, and this protective effect was further amplified on the following four days (**Figures 3B, C**). Spinal cord tissues were collected three days after surgery and microglia polarization was measured. Western blot results demonstrated that miR-148a-3p mimic decreased the expression of iNOS and increased the expression of CD206 (**Figures 3D–F**, **Supplementary Figure S4**). The results of the ELISA study were in accordance with those of the Western blot analysis, indicating that the miR-148a-3p mimic resulted in a reduction in TNF-α and an increase in IL-4 (**Figure 3G**). Additionally, immunofluorescence analysis demonstrated that the miR-148a-3p mimic decreased the co-localization of iNOS with IBA-1 and increased the co-localization of CD206 with IBA-1 (**Figures 3H, I**). These results indicated that intrathecal injection of miR-148a-3p mitigated neuropathic pain and promoted M2 polarization of microglia following SCIRI.

## 4 Discussion

The presence of neuropathic pain caused by SCIRI has a significant impact on patient outcomes. However, due to the complex mechanism, there is no recognized effective treatment. The present study demonstrated that SCIRI regulated microglial polarization, thereby inducing neuropathic pain. FOXA2/miR-148a-3p/SMURF2 feedback loop was involved in microglial polarization by regulating SIRT1 levels. Intrathecal injection of miR-148a-3p promoted the polarization of spinal microglia to M2 and alleviated neuropathic pain after SCIRI in rats.

It is well-established that microglia polarization is closely associated with neuropathic pain (48, 49). SIRT1 can influence the polarization of microglia through multiple pathways (50–52). As for how to improve the beneficial effects of SIRT1, previous studies focused on enhancing the catalytic activity of SIRT1 but ignored the SIRT1 homeostasis at the protein level (53, 54). SMURF2, a HECT-type E3 ubiquitin ligase, is known to specifically bind to SIRT1, mediating its ubiquitination and degradation in the cytoplasm (35). In this study, SCIRI-induced hyperalgesia was accompanied by increased polarization of microglia. SMURF2 was up-regulated and SIRT1 was down-regulated in spinal microglia after SCIRI. Subsequent experiments showed that SMURF2 promoted the polarization of microglia towards the M1 phenotype by reducing SIRT1 levels.

miR-148a-3p has been proven to bind with numerous target genes, playing a regulatory role in cancer, inflammation, and metabolism (55, 56). For instance, miR-148a-3p can suppress histone deacetylase 5 and enhance cancer cell invasion and migration (55). High-throughput sequencing reveals that elevated expression of miR-148a-3p in human umbilical cord mesenchymal stem cells may foster the polarization of M2 macrophages, thereby potentially mitigating the severity of osteoarthritis (57). To explore the upstream mechanism of SMURF2, we predicted and subsequently confirmed that SMURF2 is a target gene for miR-148a-3p. The results showed that miR-148a-3p inhibited SIRT1 ubiquitination degradation by targeting SMURF2 and promoted microglia polarization toward M2.

**Figure 7:**
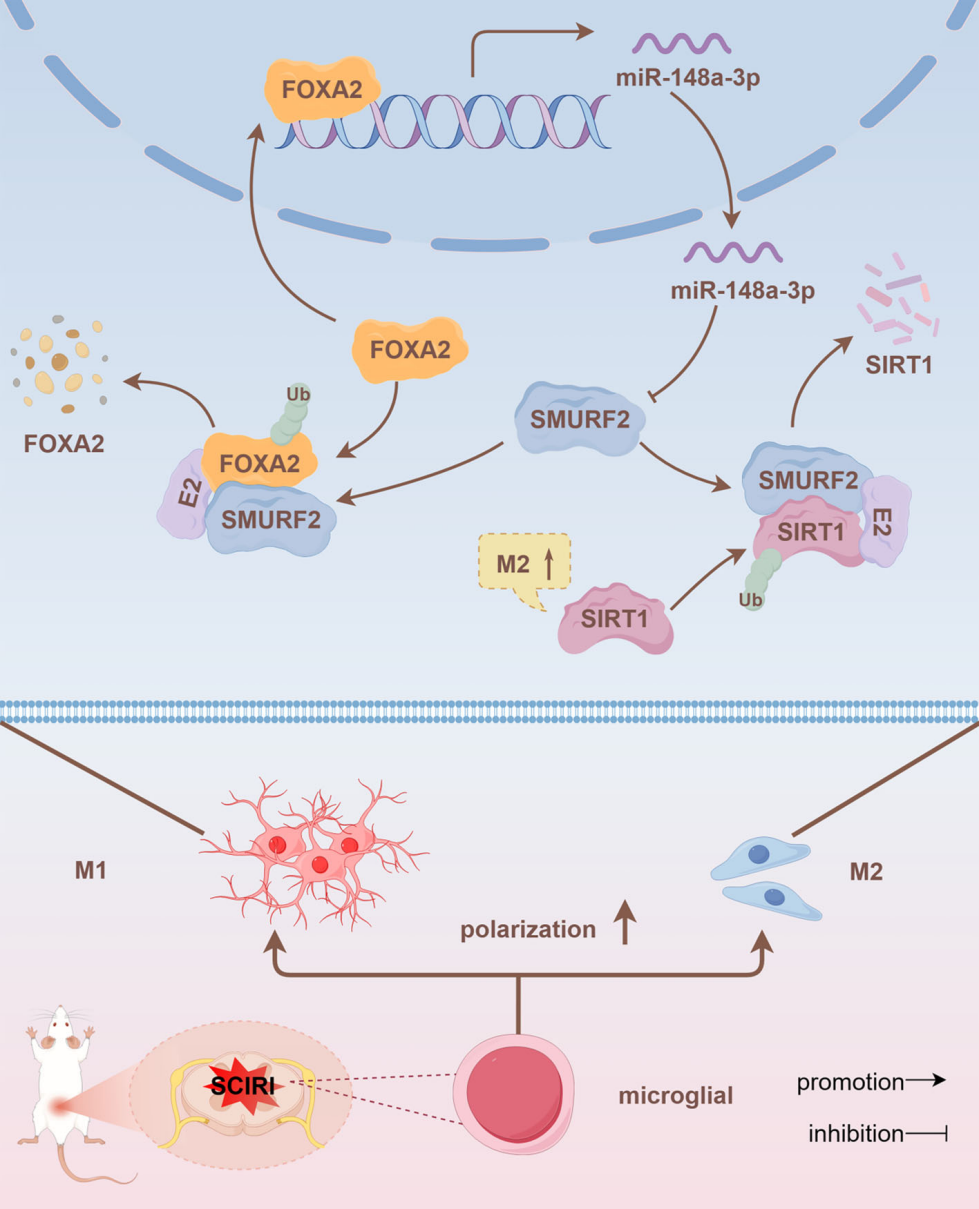
FOXA2/miR-148a-3p/SMURF2 feedback loop regulates SIRT1 ubiquitination, affecting microglia polarization and thereby regulating neuropathic pain.

It is noteworthy that our findings indicated that SMURF2 could affect miR-148a-3p expression. Therefore, we postulated that SMURF2 functions as an E3 ubiquitin ligase to regulate upstream miR-148a-3p, thereby reversing the regulation of miR-148a-3p. By predicting miRNA transcription factors and SMURF2 ubiquitination substrates, we focused on FOXA2. Subsequent experiments confirmed that FOXA2, as a transcription factor, transcriptionally regulated miR-148a-3p. Additionally, it was found that SMURF2 could bind to FOXA2 and promote FOXA2 ubiquitination and degeneration. Further animal experiments demonstrated that intrathecal injection of miR-148a-3p could effectively polarize spinal microglia to the M2 type and alleviate neuropathic pain (**Figure 7**).

Recent studies have established miRNAs as promising diagnostic and prognostic biomarkers across various diseases (58–60), while extracellular vesicle (EV)-based delivery systems have demonstrated significant therapeutic potential for targeted interventions (61). For example, erythrocyte-derived EVs effectively delivered anti-miR-214 oligonucleotides to suppress pathological bone resorption (62), and CD33-targeted EVs carrying miR-125b antisense oligonucleotides enhanced leukemia stem cell clearance (63). Building on these advancements, future investigations should explore the dual utility of engineered EVs for both targeted delivery of miR-148a-3p to modulate neuropathic pain through microglial polarization and concurrent development as companion diagnostic biomarkers for neurological disorders. While the study established the FOXA2/miR-148a-3p/SMURF2 axis as a critical regulator of microglial polarization, it is imperative to acknowledge that this regulatory network may be influenced by numerous additional factors. Notably, prior work has shown that miR-148a-3p is involved in the lncRNA-H19/Rock2 axis in regulating oxidative stress in myocardial ischemia-reperfusion injury (64) and targeted PTEN to amplify proinflammatory responses (65). These findings suggest that the FOXA2/miR-148a-3p/SMURF2 axis may be dynamically regulated by multiple factors, and future research should focus on these interactions network between multiple upstream and downstream factors of miR-148a-3p to deepen the understanding of the mechanisms underlying neuropathic pain.

## 5 Conclusion

FOXA2/miR-148a-3p/SMURF2 signaling feed-forward loop is involved in SIRT1 ubiquitination and microglia polarization, regulating SCIRI-induced neuropathic pain. Intrathecal administration targeting miR-148a-3p represents a promising therapeutic strategy for clinical intervention in neuropathic pain by reversing the dysregulation of this signaling axis.

## Data Availability

The original contributions presented in the study are included in the article/**Supplementary Material**. Further inquiries can be directed to the corresponding author/s.
