# Supplementary material for: FOXA2/miR-148a-3p/SMURF2 signaling feed-forward loop alleviates spinal cord ischemia-reperfusion injury-induced neuropathic pain by modulating microglia polarization in rats
Source: Front Immunol. 2025 May 13;16:1563377. doi: 10.3389/fimmu.2025.1563377 (PMC12108549; doi:10.3389/fimmu.2025.1563377)
Supplement: Supplementary file 1 [file DataSheet1.docx]

Supplementary Material

**Table S1** Primers used for RT-qPCR and ChIP-qPCR

| Primer | Forward | Reverse |
| --- | --- | --- |
| SIRT1 | ACGCCTTATCCTCTAGTTCCTGTGG | CGGTCTGTCAGCATCATCTTCCAAG |
| SMURF2 | CAATGCCATCAATCGCCTCAAAGAC | CAACTTGTCCTCCCGTGCCTATTC |
| FOXA2 | GCACCTGAGTCCGAGTCTGAG | CCCGCCTGCCCGTACATAG |
| GAPDH | GACATGCCGCCTGGAGAAAC | AGCCCAGGATGCCCTTTAGT |
| U6 | GCTCGCTTCGGCAGCACA | GAGGTATTCGCACCAGAGGA |
| miR-148a-3p | CCGCTCAGTGCACTACAGAACTTTG |  |
| miR-152-3p | GCTCAGTGCATGACAGAACTTGG |  |
| miR-148b-3p | GCTCAGTGCATCACAGAACTTTGT |  |
| miR-542-3p | GCGCCTGTGACAGATTGATAACTGAAA |  |
| miR-223-3p | CCGCTGTCAGTTTGTCAAATACCCC |  |
| pri-miR-148a | TGAGTTAGCTGCTGGCAAAA | TTTTAACAGCCCGATTCGAC |

**
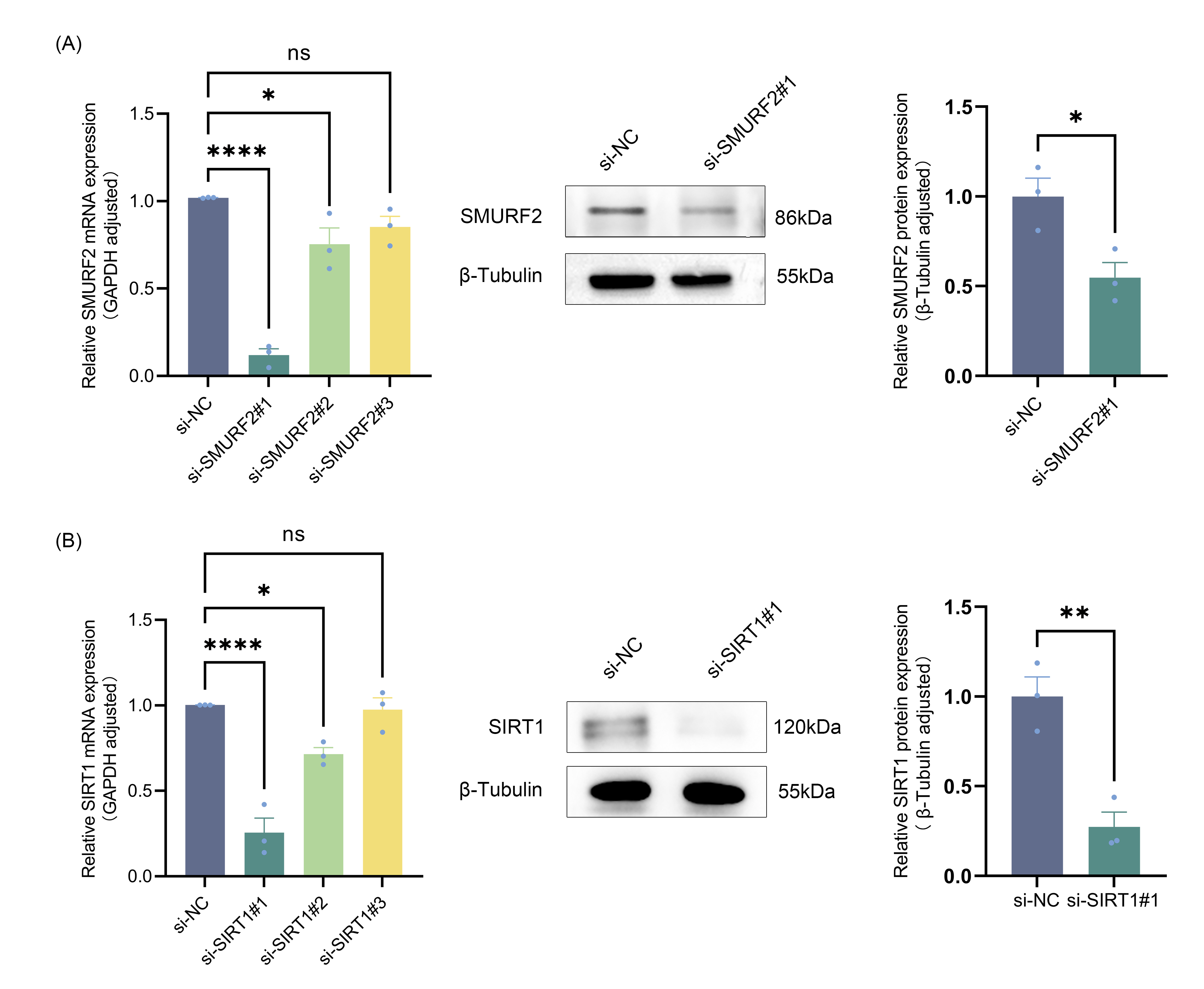
**

**Supplementary Figure S1.** Knockout efficiency of SMURF2 and SIRT1. (A) RT-qPCR and Western blot were used to verify the knockdown efficiency of SMURF2. (B) RT-qPCR and Western blot were used to verify the knockdown efficiency of SIRT1. Unpaired t-test and one-way ANOVA were utilized. Significance levels: *P<0.05, **P<0.01, ****P<0.0001.

**
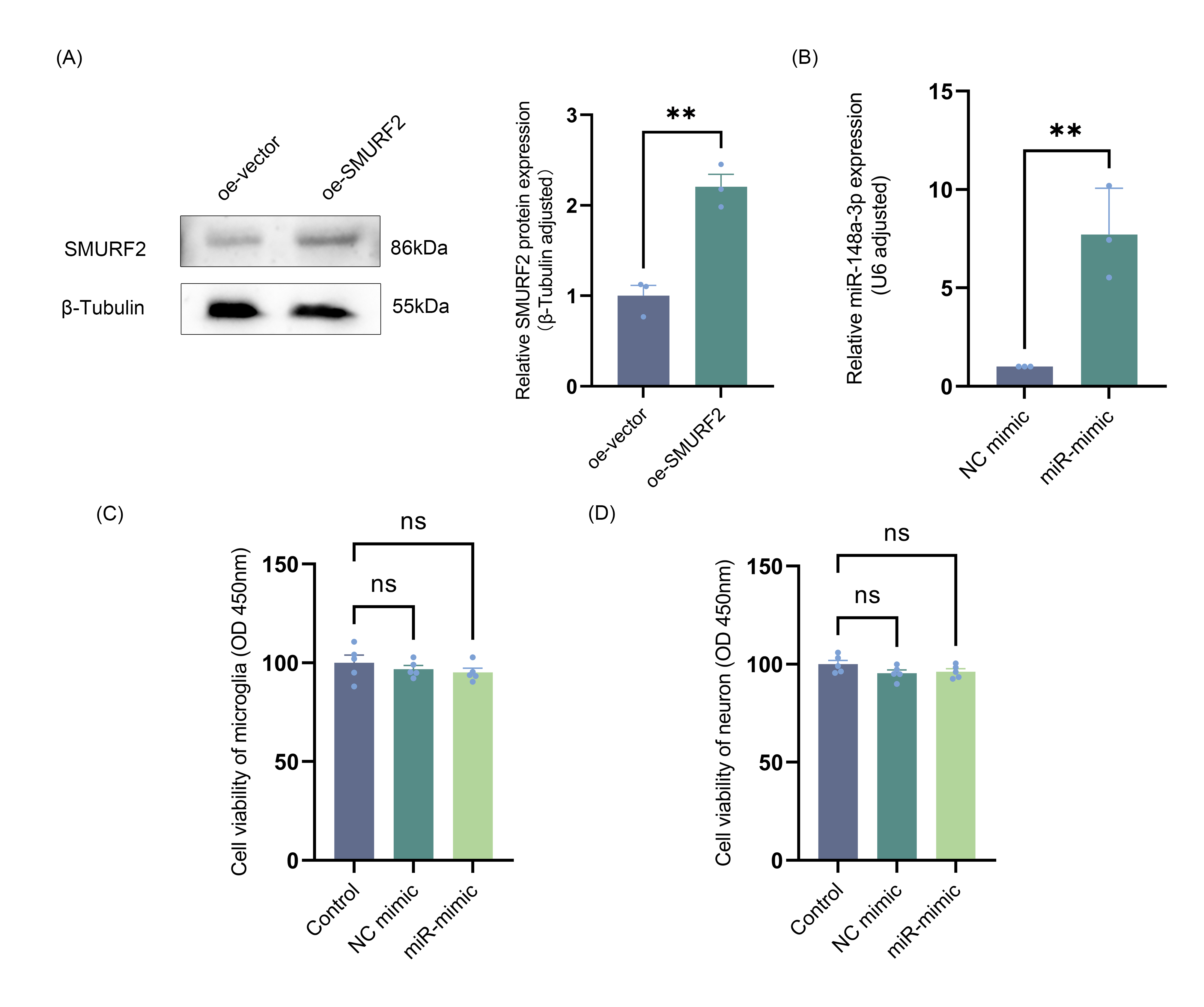
**

**Supplementary Figure S2.** Validation of SMURF2 overexpression efficiency and miR-mimic overexpression in vitro, and examination of miR-mimic’s effects on microglial and neuronal cell viability. (A) Overexpression efficiency of SMURF2. (B) RT-qPCR analysis was employed to verify the overexpression efficiency of miR-148b-3p in HAPI cells. (C) Proliferation of HAPI cells was evaluated by CCK8 assay. (D) Proliferation of VSC4.1 cells was evaluated by CCK8 assay. Unpaired t-test and one-way ANOVA were utilized. Significance levels: **p < 0.01.

**
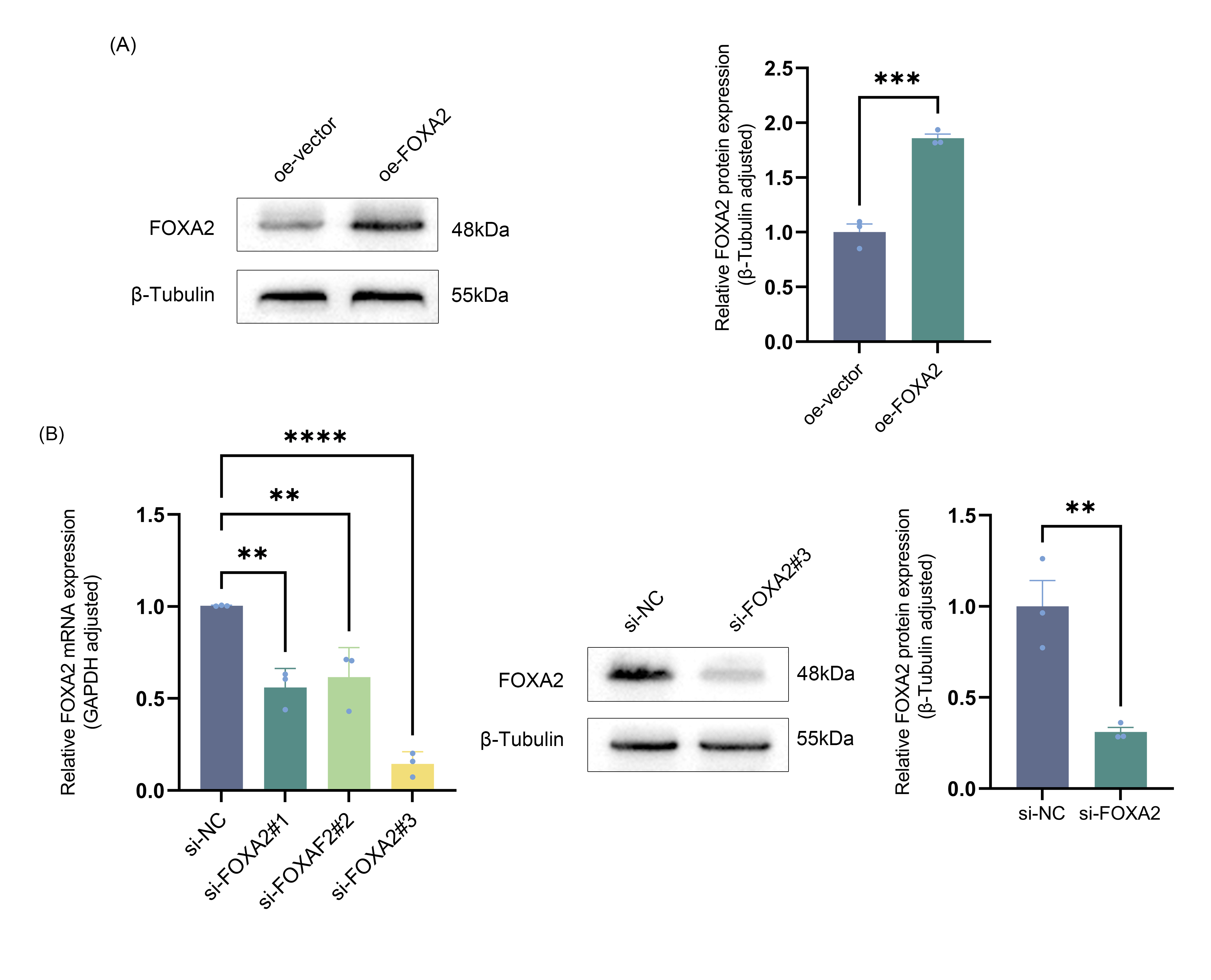
**

**Supplementary Figure S3.** Validation of FOXA2 overexpression and knockdown efficiency. (A) Representative Western blot of FOXA2 and quantitative protein analysis of FOXA2. (B) RT-qPCR and Western blot analysis were employed to verify the knockout efficiency of FOXA2. Unpaired t-test and one-way ANOVA were utilized. Significance levels: **P<0.01, ***P<0.001, ****P<0.0001.

**
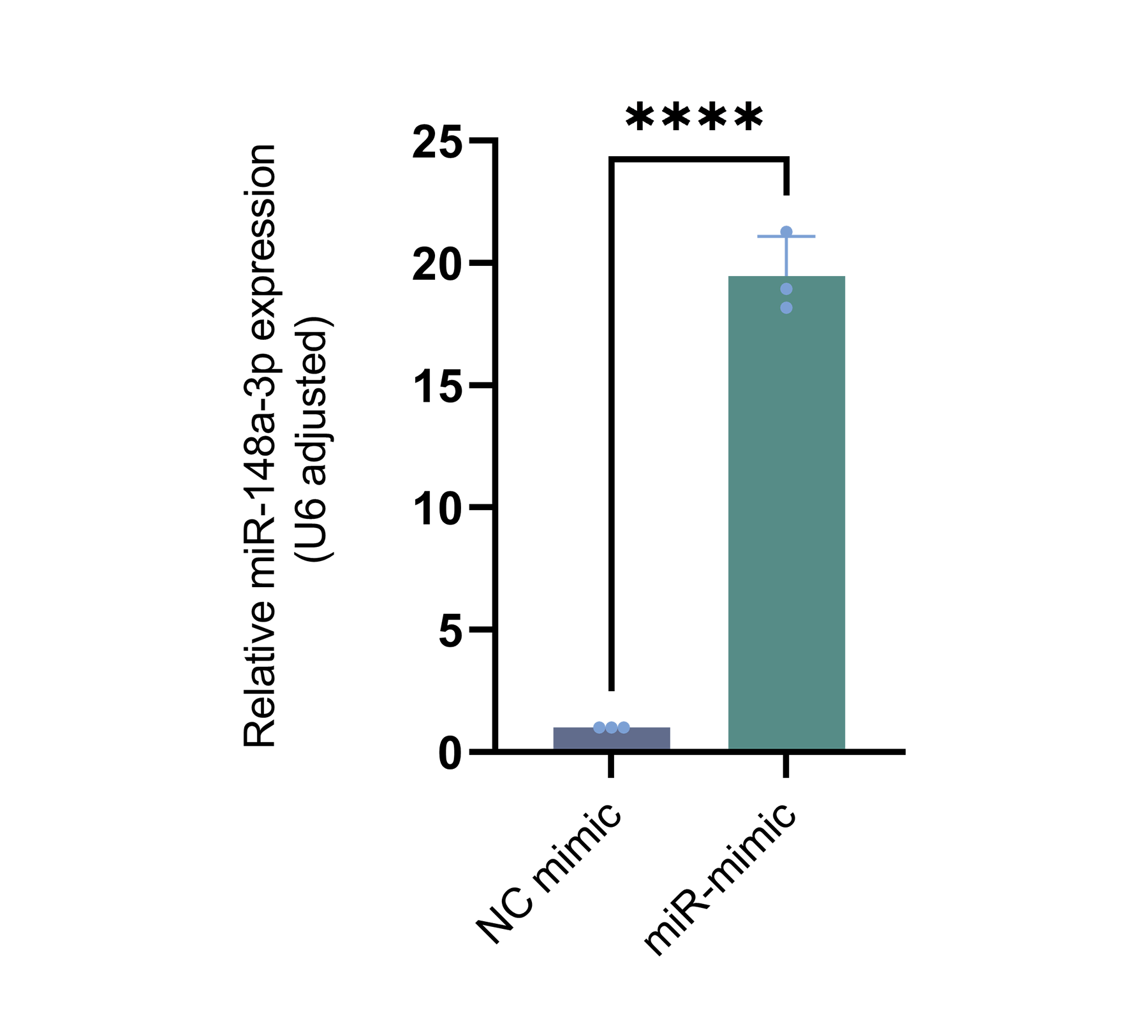
**

**Supplementary Figure S4.** In vivo validation of miR-mimic overexpression efficiency.Unpaired t-test were utilized. Significance levels: ****P<0.0001.
