# Supplementary material for: FOXA2/miR-148a-3p/SMURF2 signaling feed-forward loop alleviates spinal cord ischemia-reperfusion injury-induced neuropathic pain by modulating microglia polarization in rats
Source: Front Immunol. 2025 May 13;16:1563377. doi: 10.3389/fimmu.2025.1563377 (PMC12108549; doi:10.3389/fimmu.2025.1563377)

Figure1C

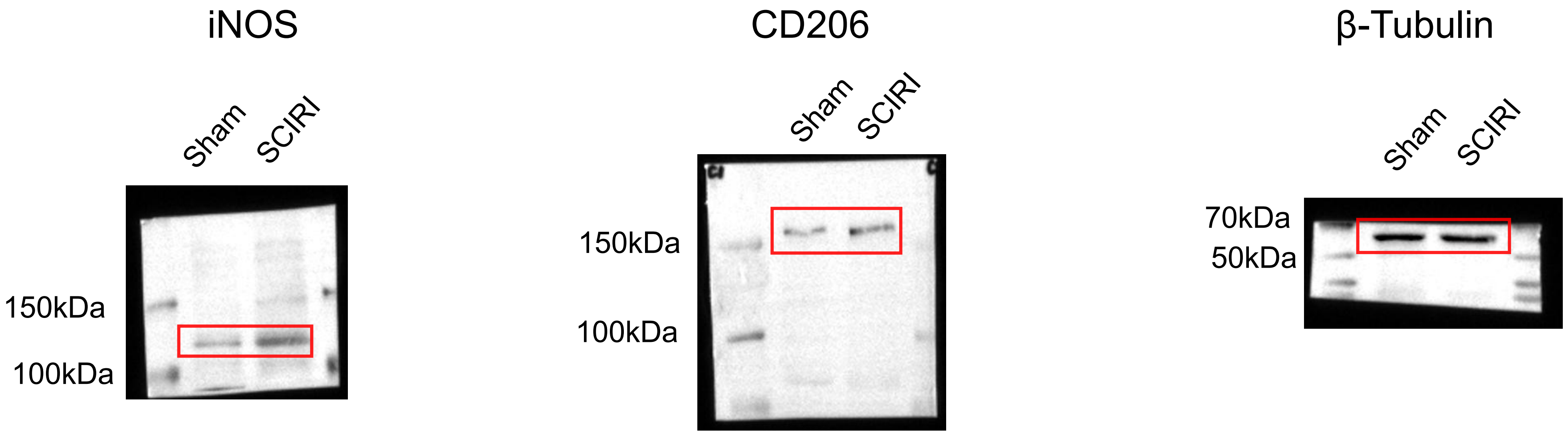
iNOS
Sham
SCIRI
150kDa
100kDa
CD206
Sham
SCIRI
150kDa
100kDa
β-Tubulin
Sham
SCIRI
70kDa
50kDa

Figure1F

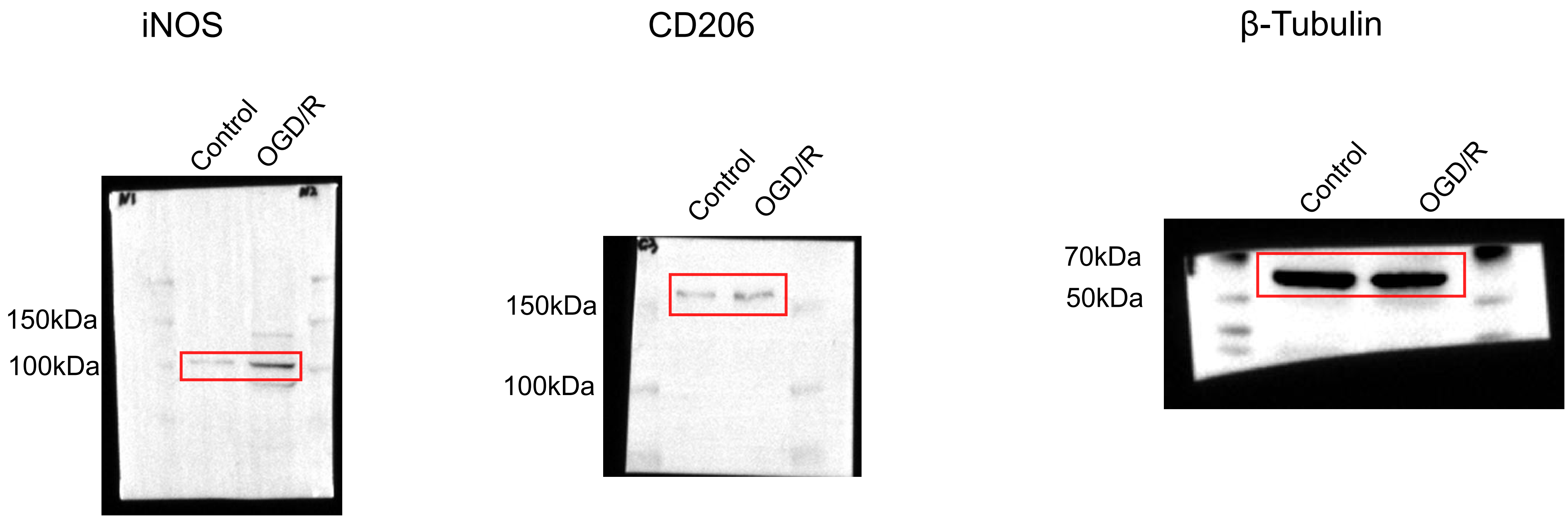

iNOS
Control
OGD/R
150kDa
100kDa
CD206
Control
OGD/R
150kDa
100kDa
β-Tubulin
Control
OGD/R
70kDa
50kDa

Figure2A

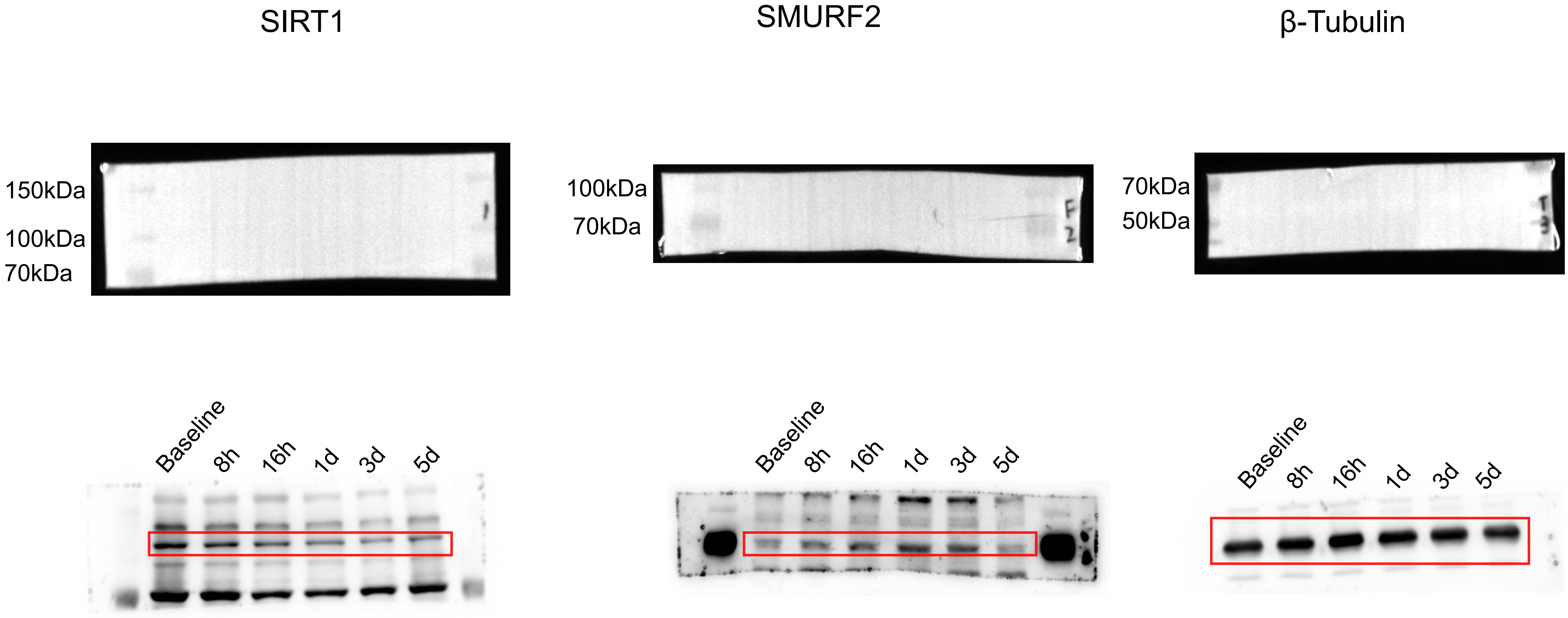
SIRT1
SMURF2
β-Tubulin
150kDa
100kDa
70kDa
100kDa
70kDa
70kDa
50kDa
Baseline
8h
16h
1d
3d
5d

Figure2F

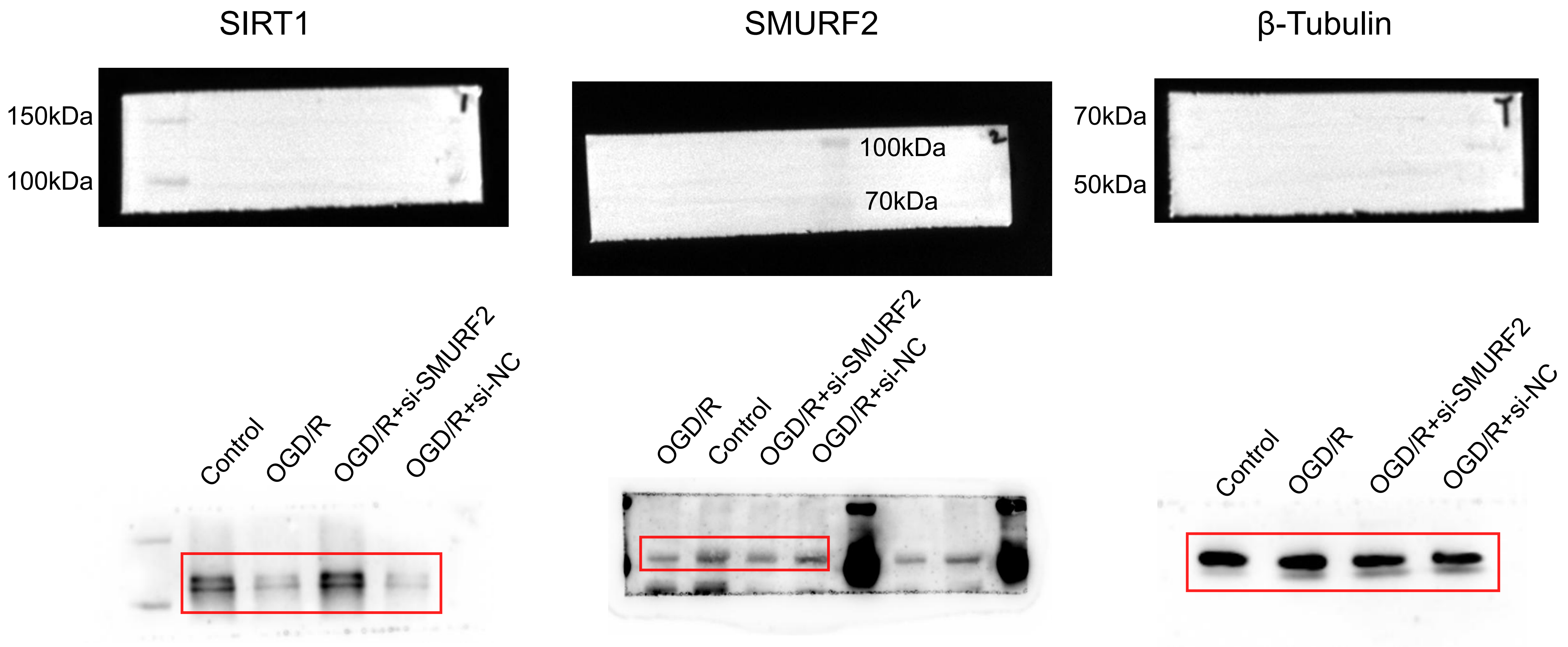

SIRT1
SMURF2
β-Tubulin
150kDa
100kDa
100kDa
70kDa
70kDa
50kDa
Control
OGD/R
OGD/R+si-SMURF2
OGD/R+si-NC
OGD/R
Control
OGD/R+si-SMURF2
OGD/R+si-NC
Control
OGD/R
OGD/R+si-SMURF2
OGD/R+si-NC

Figure2I

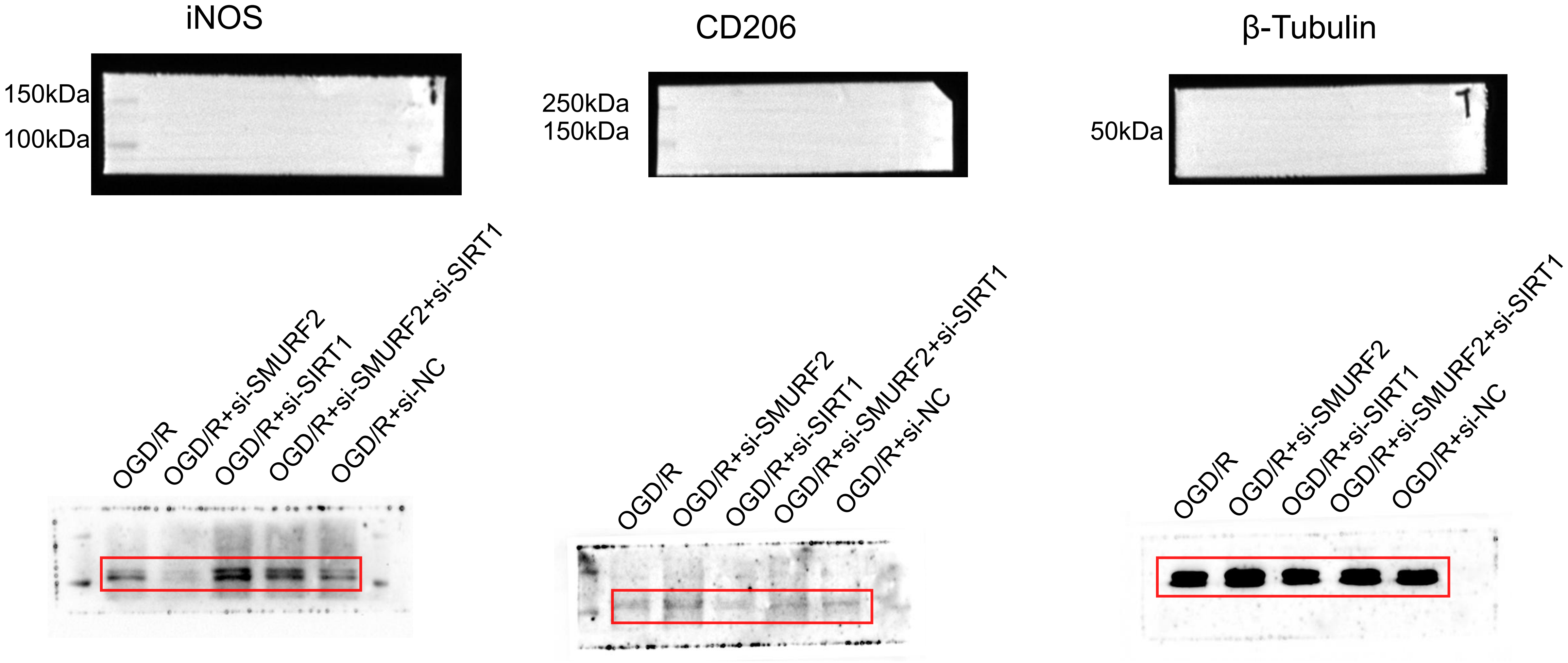
iNOS
150kDa
100kDa
CD206
250kDa
150kDa
β-Tubulin
50kDa
OGD/R
OGD/R+si-SMURF2
OGD/R+si-SIRT1
OGD/R+si-SMURF2+si-SIRT1
OGD/R+si-NC

Figure3E

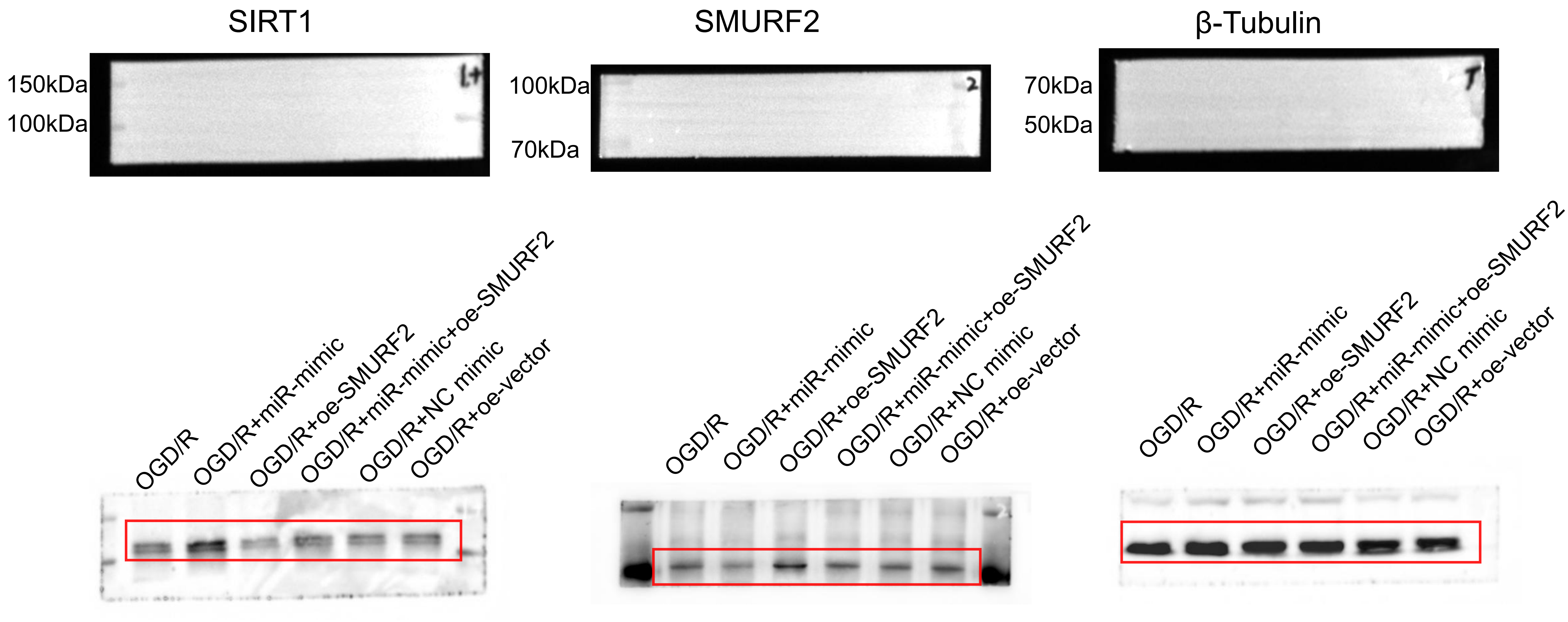
SIRT1
SMURF2
β-Tubulin
150kDa
100kDa
100kDa
70kDa
70kDa
50kDa
OGD/R
OGD/R+miR-mimic
OGD/R+oe-SMURF2
OGD/R+miR-mimic+oe-SMURF2
OGD/R+NC mimic
OGD/R+oe-vector

Figure3H

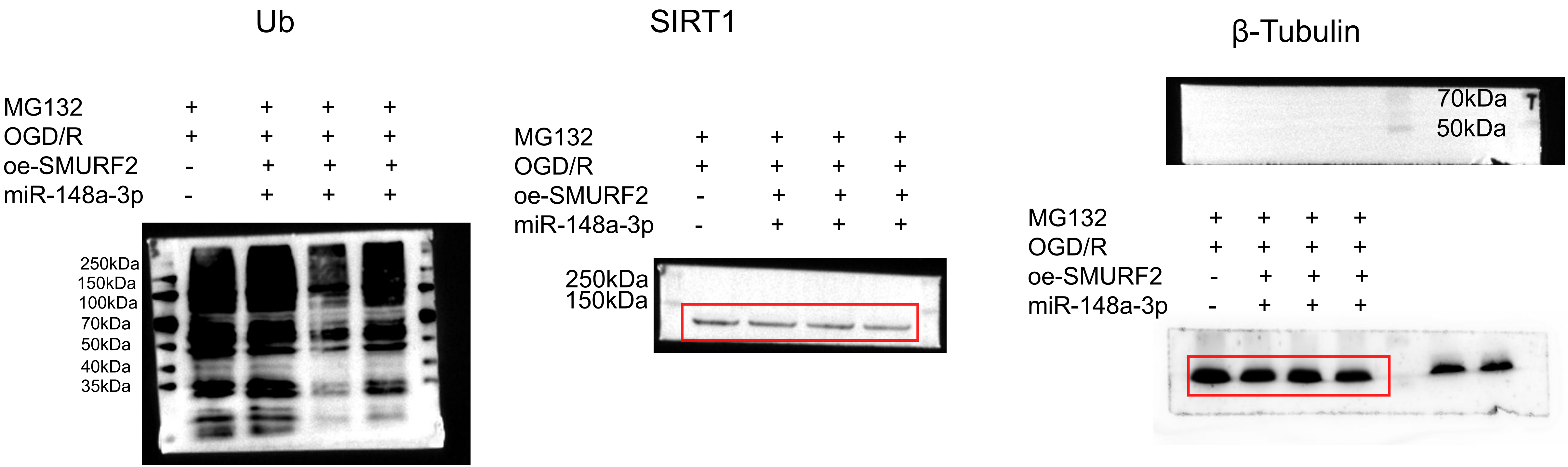
Ub
MG132 + + + +
OGD/R + + + +
oe-SMURF2 - + + +
miR-148a-3p - + + +
250kDa
150kDa
100kDa
70kDa
50kDa
40kDa
35kDa
SIRT1
MG132 + + + +
OGD/R + + + +
oe-SMURF2 - + + +
miR-148a-3p - + + +
250kDa
150kDa
β-Tubulin
70kDa
50kDa
MG132 + + + +
OGD/R + + + +
oe-SMURF2 - + + +
miR-148a-3p - + + +

Figure4A

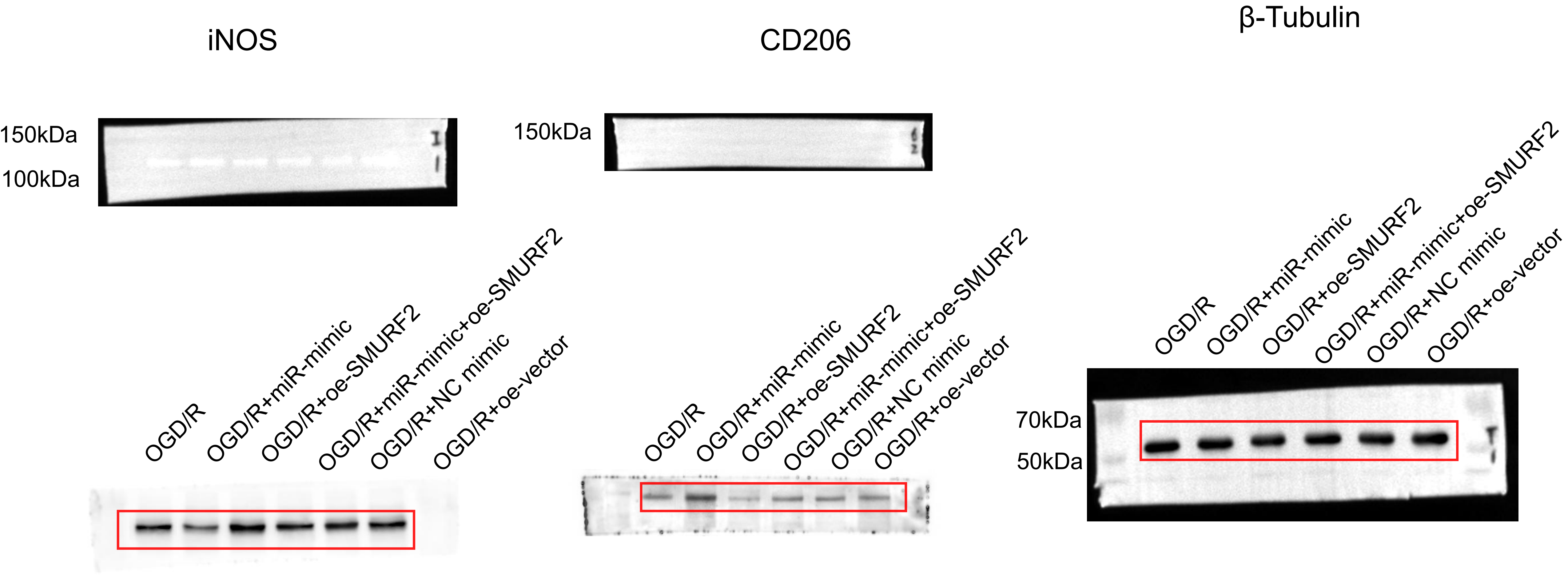
iNOS
CD206
β-Tubulin
150kDa
100kDa
150kDa
70kDa
50kDa
OGD/R
OGD/R+miR-mimic
OGD/R+oe-SMURF2
OGD/R+miR-mimic+oe-SMURF2
OGD/R+NC mimic
OGD/R+oe-vector

Figure5B

FOXA2

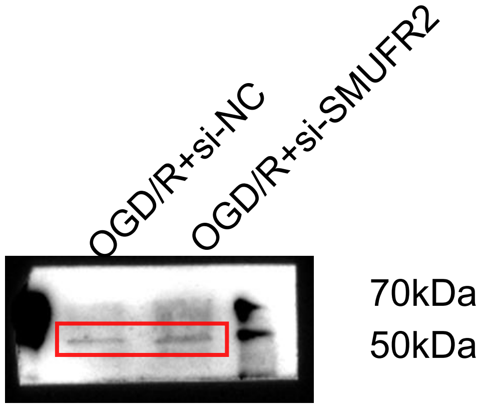

β-Tubulin

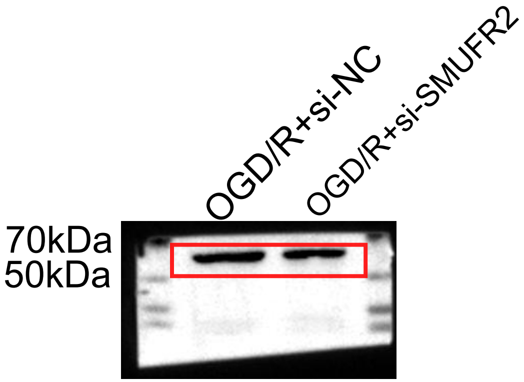

Figure5C

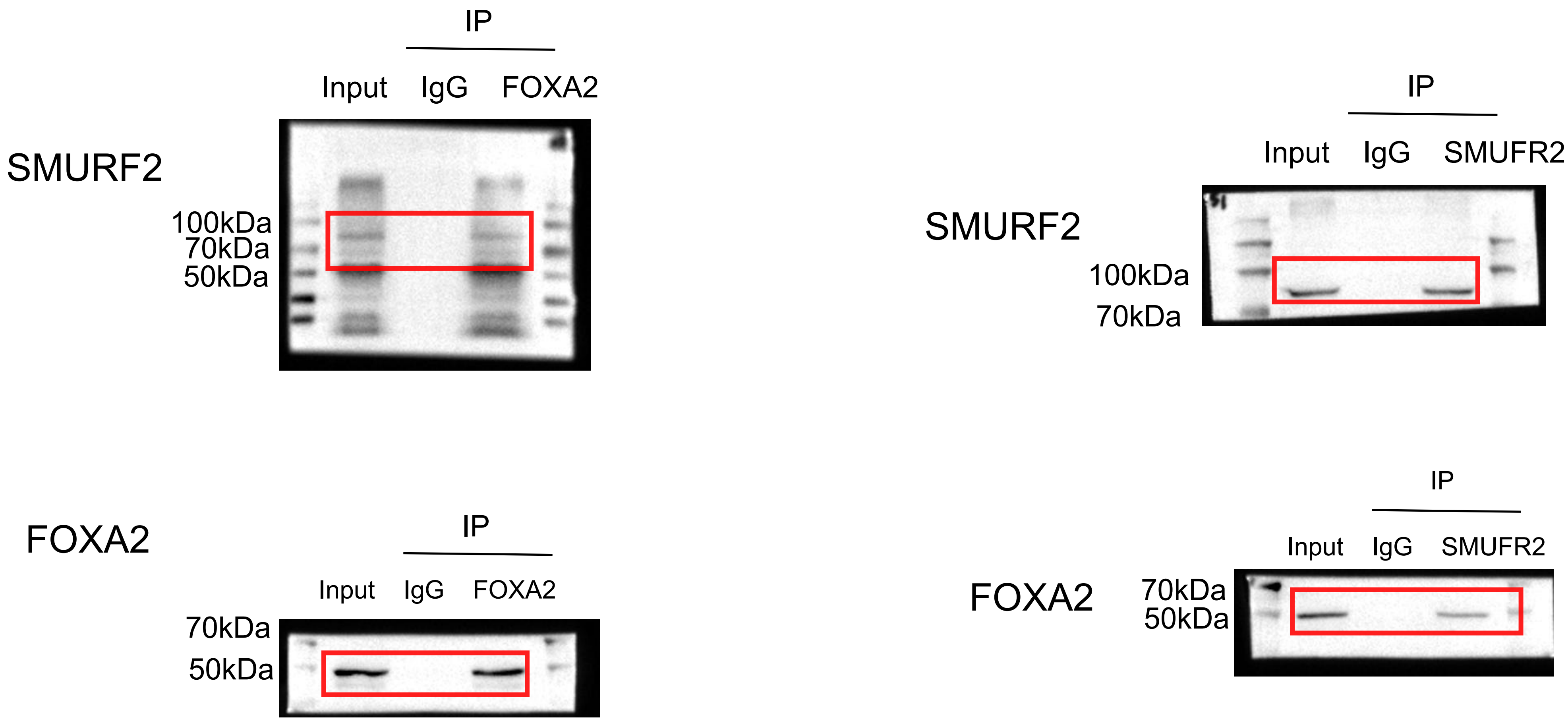

SMURF2
IP
Input
IgG
FOXA2
100kDa
70kDa
50kDa
SMURF2
IP
Input
IgG
SMUFR2
100kDa
70kDa
FOXA2
IP
Input
IgG
FOXA2
70kDa
50kDa
FOXA2
IP
Input
IgG
SMUFR2
70kDa
50kDa

Figure5D

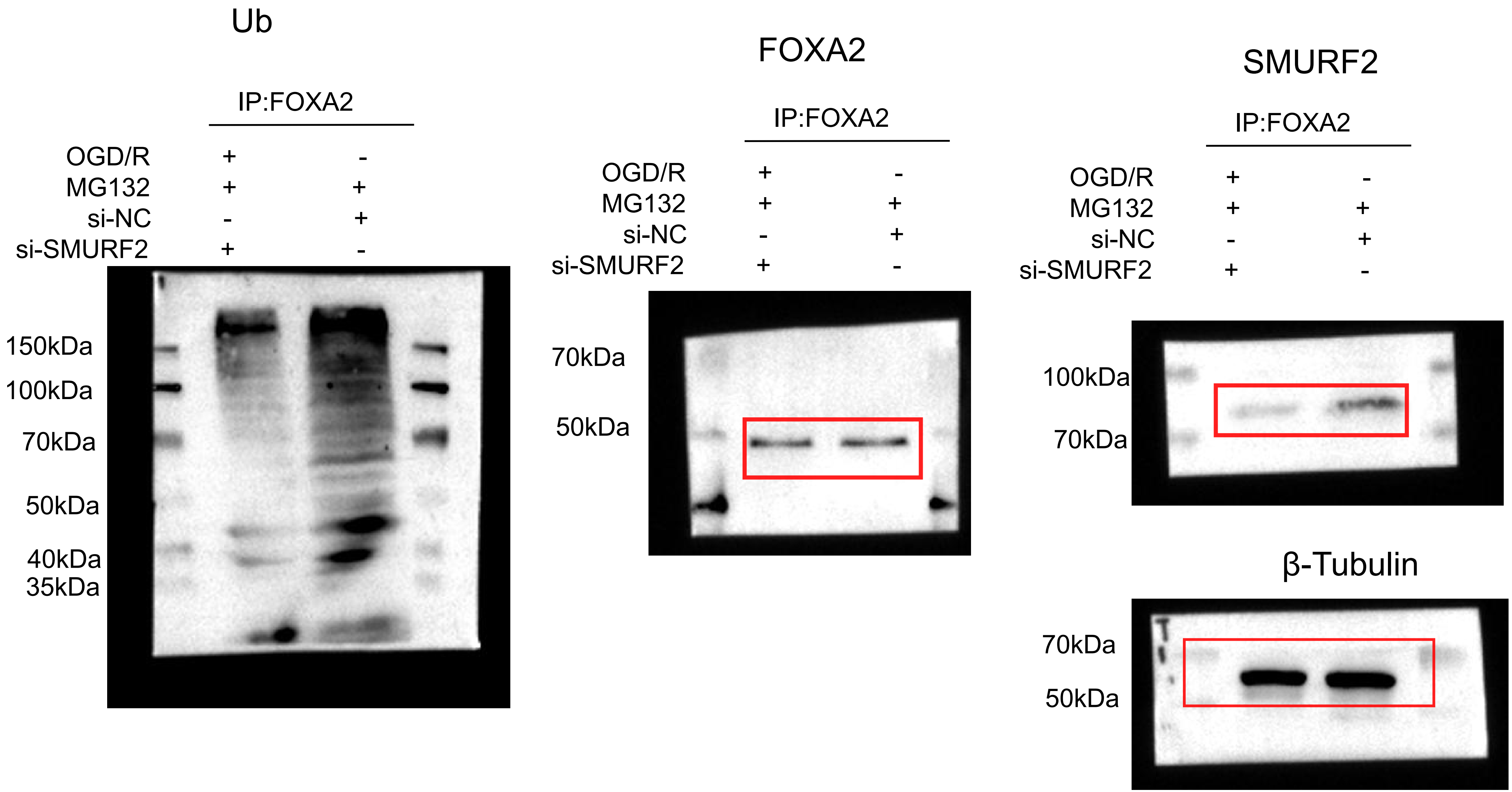
Ub
IP:FOXA2
OGD/R + -
MG132 + +
si-NC - +
si-SMURF2 + -
150kDa
100kDa
70kDa
50kDa
40kDa
35kDa
FOXA2
IP:FOXA2
OGD/R + -
MG132 + +
si-NC - +
si-SMURF2 + -
70kDa
50kDa
SMURF2
IP:FOXA2
OGD/R + -
MG132 + +
si-NC - +
si-SMURF2 + -
100kDa
70kDa
β-Tubulin
70kDa
50kDa

Figure5E

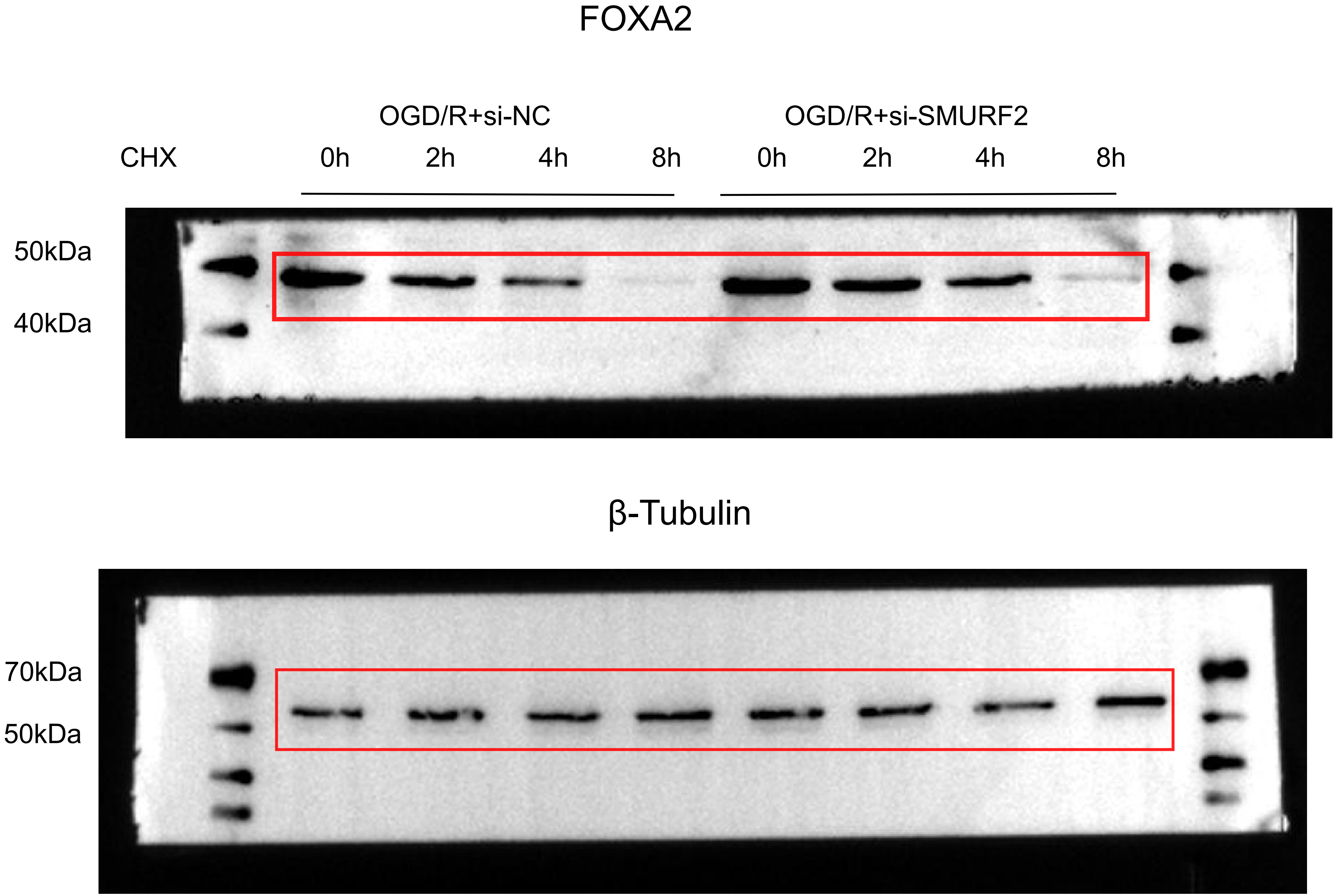
FOXA2
OGD/R+si-NC
OGD/R+si-SMURF2
CHX
0h
2h
4h
8h
0h
2h
4h
8h
50kDa
40kDa
β-Tubulin
70kDa
50kDa

Figure5F

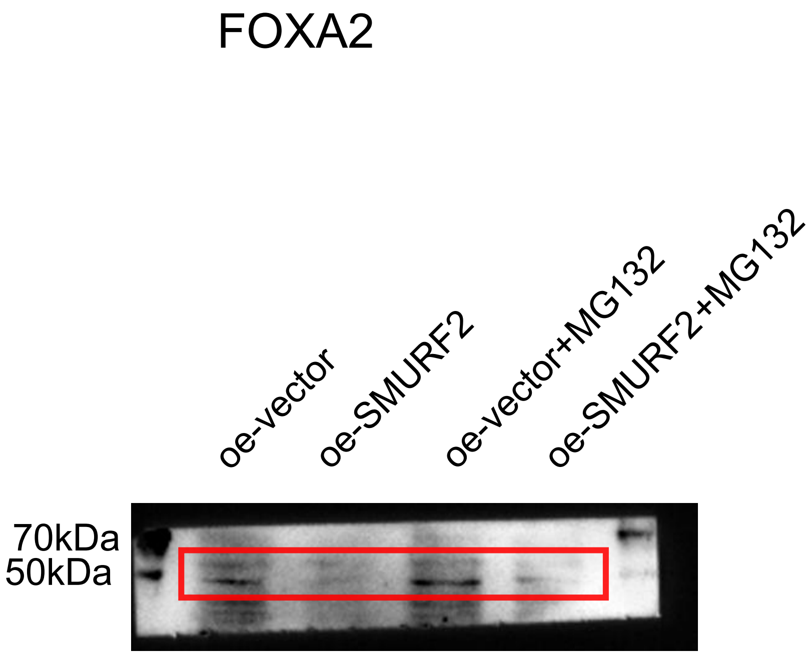
FOXA2
oe-vector
oe-SMURF2
oe-vector+MG132
oe-SMURF2+MG132
70kDa
50kDa

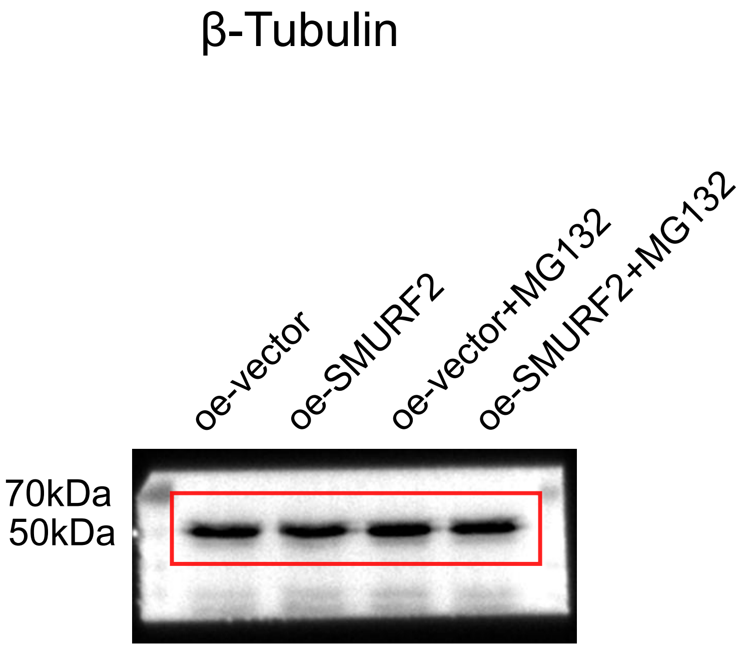
β-Tubulin
oe-vector
oe-SMURF2
oe-vector+MG132
oe-SMURF2+MG132
70kDa
50kDa

Figure6C

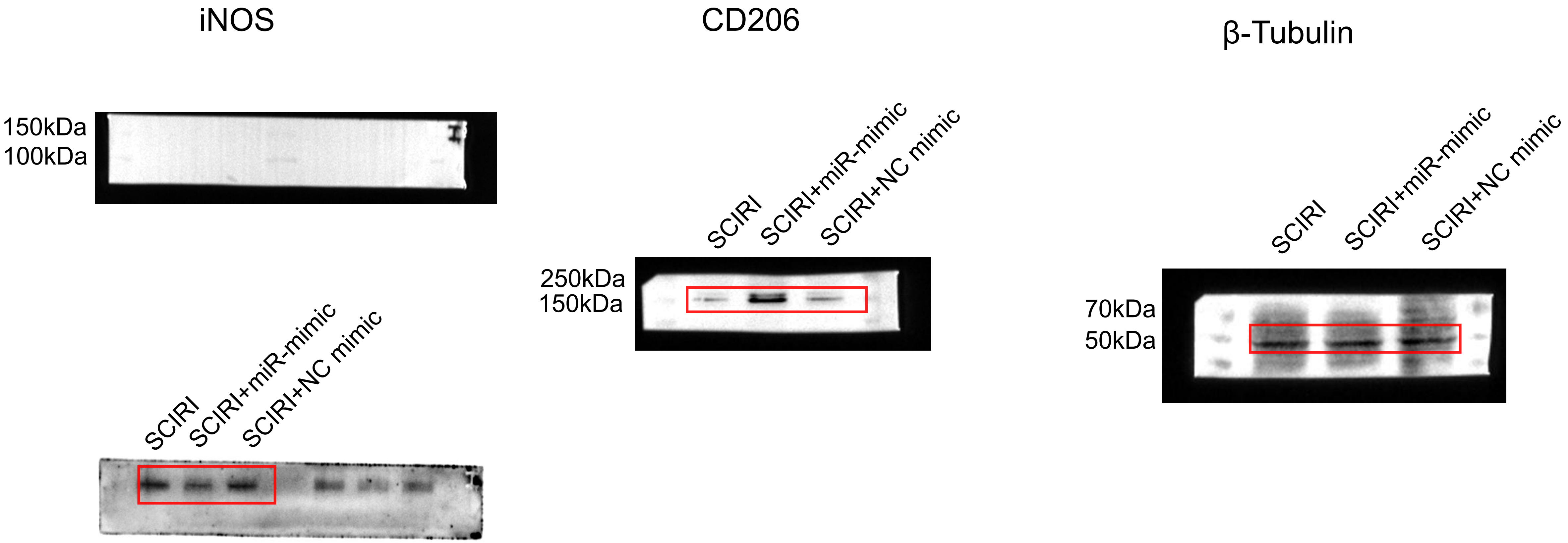
iNOS
150kDa
100kDa
SCIRI
SCIRI+miR-mimic
SCIRI+NC mimic
CD206
SCIRI
SCIRI+miR-mimic
SCIRI+NC mimic
250kDa
150kDa
β-Tubulin
SCIRI
SCIRI+miR-mimic
SCIRI+NC mimic
70kDa
50kDa

Supplementary Figure S1 A SMURF2 and β-Tubulin
Figure S1 B SIRT1 and β-Tubulin

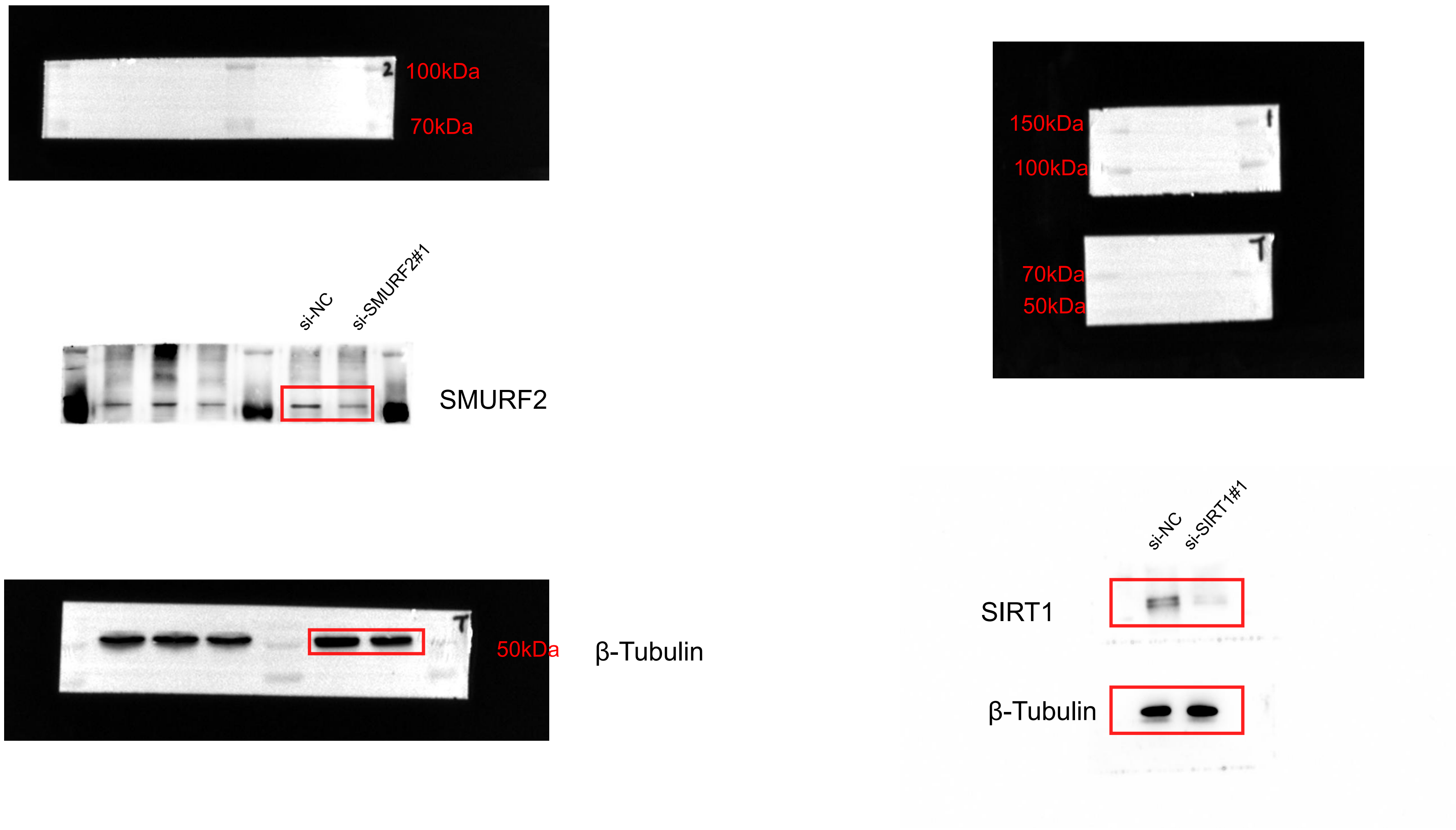

Supplementary Figure S2 A SMURF2 and β-Tubulin

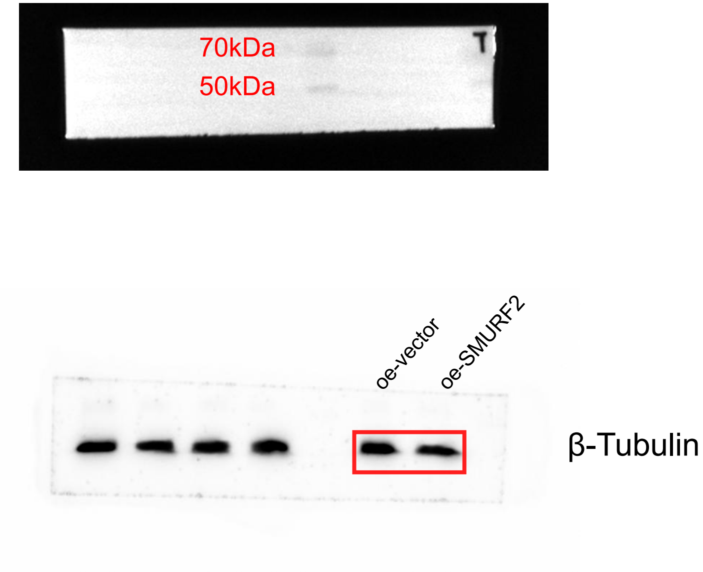

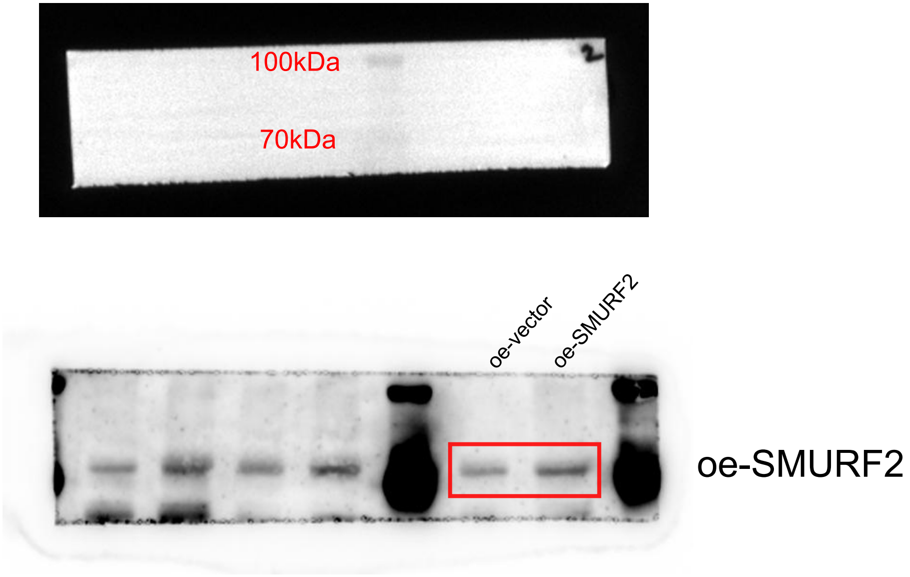

Supplementary Figure S3 A FOXA2 and β-Tubulin
Figure S3 B FOXA2 and β-Tubulin

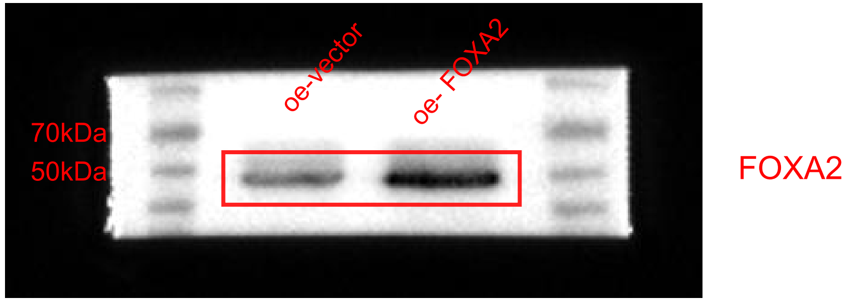

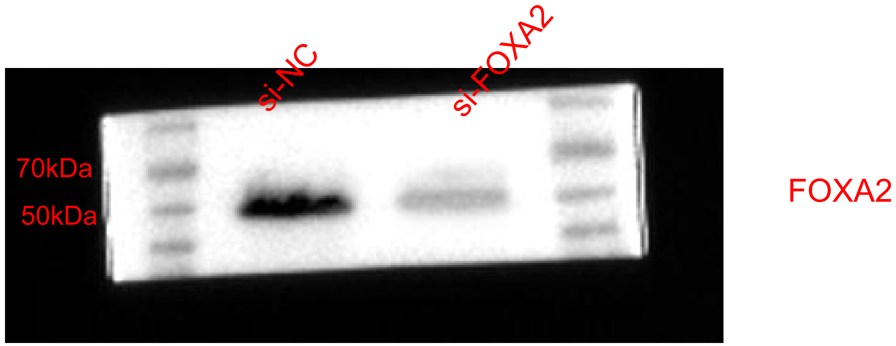

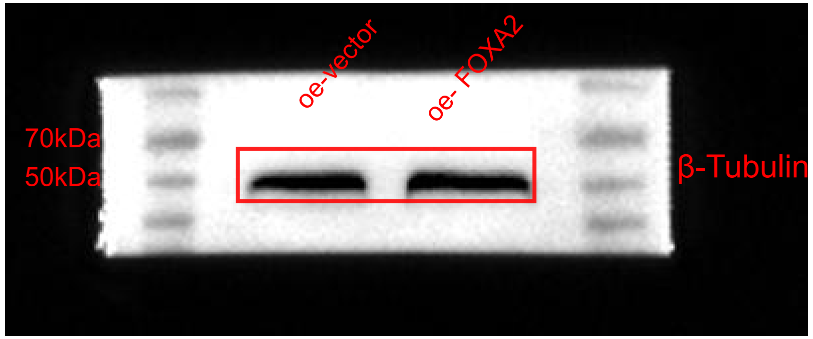

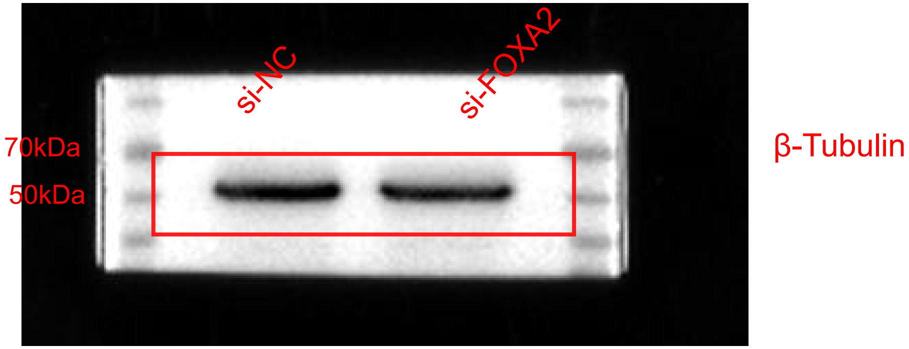

Supplement: Supplementary file 2 [file DataSheet2.pdf]
